# Specificities of chemosensory receptors in the human gut microbiota

**DOI:** 10.1073/pnas.2508950122

**Published:** 2025-08-26

**Authors:** Wenhao Xu, Ekaterina Jalomo-Khayrova, Vadim M. Gumerov, Patricia A. Ross, Tania S. Köbel, Daniel Schindler, Gert Bange, Igor B. Zhulin, Victor Sourjik

**Affiliations:** ^a^Max Planck Institute for Terrestrial Microbiology, Marburg 35043, Germany; ^b^Center for Synthetic Microbiology, Marburg 35043, Germany; ^c^Department of Chemistry, Philipps-University Marburg, Marburg 35043, Germany; ^d^Department of Microbiology and Translational Data Analytics Institute, The Ohio State University, Columbus, OH 43210

**Keywords:** signal transduction, gut microbiome, sensory domains, metabolites, chemotaxis

## Abstract

The human gut is rich in metabolites and harbors a complex microbial community, yet surprisingly little is known about the spectrum of chemical signals detected by the large variety of sensory receptors present in the gut microbiome. Here, we systematically mapped the ligand specificities of selected extracytoplasmic sensory domains from twenty members of the human gut microbiota, with a primary focus on the abundant and physiologically important class of Clostridia. Twenty-five metabolites from different chemical classes—including amino acids, nucleobase derivatives, amines, indole, and carboxylates—were identified as specific ligands for fifteen sensory domains from nine bacterial species, which represent all three major functional classes of transmembrane receptors: chemotaxis receptors, histidine kinases, and enzymatic sensors. We have further characterized the specificity and evolution of ligand binding to Cache superfamily sensors specific for lactate, dicarboxylic acids, and for uracil and short-chain fatty acids (SCFAs). Structural and biochemical analysis of the dCache sensor of uracil and SCFAs revealed that its two different ligand types bind at distinct sensory modules. Overall, combining experimental identification with computational analyses, we were able to assign ligands to approximately half of the Cache-type chemotaxis receptors found in the eleven gut commensal genomes from our set, with carboxylic acids representing the largest ligand class. Among these, the most commonly found ligand specificities were for lactate and formate, indicating a particular importance of these metabolites in the human gut microbiota and consistent with their observed growth-promoting effects on selected bacterial commensals.

## Significance

Environmental sensing is highly important for bacterial growth and survival in diverse habitats, but the sensory repertoire of bacterial receptors remains largely uncharacterized. While most previous studies have focused on individual model organisms, here we took a habitat-centric approach, systematically identifying chemically diverse ligands of selected Cache-type domains from a set of nonmodel bacteria inhabiting a particular ecological niche, the human gut. Our analyses provided important insights into the sensory and metabolic preferences of motile commensal bacteria in the human gut microbiota, indicating the prevalence of cross-feeding mediated by short-chain carboxylic acids. Furthermore, we biochemically and structurally characterized several specificities of Cache domains and demonstrated a surprisingly high evolvability of these sensors toward metabolites relevant to the gut microbiota.

The gut microbiota, a complex and dynamic community of microorganisms residing in the human gastrointestinal (GI) tract, plays an important role in host health (1, 2). The composition and resilience of the gut microbiota largely depend on the complex metabolic and signaling interactions of microorganisms with the host and among each other (2, 3), which requires the ability to detect changes in the levels of nutrients and signaling molecules (4, 5) using a variety of signal transduction systems (6). Major functional families of bacterial environmental sensors include chemotaxis receptors (methyl-accepting chemotaxis proteins, or MCPs), which control bacterial motility in environmental gradients (7, 8); histidine kinases (HKs), which mediate transcriptional responses (9); and enzymatic sensors, such as adenylate, deadenylate, and diguanylate cyclases (DGCs) and corresponding phosphodiesterases, which regulate levels of second messengers (10). However, although the mechanisms of signal transduction through these pathways have been extensively studied, much less is known about the specificities of their sensory receptors (6, 7, 11).

Canonical sensory receptors possess extracytoplasmic ligand-binding domains (LBDs), although various alternative modes of sensory perception have also been described (7, 11, 12). LBDs are structurally highly diverse (7) and function as independent ligand-binding modules that can be shared by different sensor families (13). Although LBDs can be well annotated bioinformatically, their ligand specificity is highly variable and rapidly evolving, and in most cases cannot be predicted from the LBD sequence or structure alone (7, 14). The reported repertoire of known signals perceived by sensory receptors across bacterial species is remarkably broad and organism-specific, including various nutritionally valuable metabolites, bacterial and eukaryotic signaling molecules, as well as ions, gases, pH, light, and various environmental stresses (15, 16). These sensory capabilities of bacteria are known to be closely linked to their living environment (17, 18), but which of the numerous host- and bacteria-derived metabolites in the human gut (19, 20) serve as preferred signals for gut bacteria remains unknown.

To systematically identify these signals, we selected approximately one hundred representative LBDs from the previously reported dataset of extracytoplasmic sensory domains derived from the human gut microbiome (21), of which seventy proved amenable to subsequent biochemical analysis. We focused on bacteria from the class of Clostridia due to their abundance in the gut microbiota and their critical role in the maintenance of gut homeostasis (22). In addition, this class contains the most abundant chemotactic bacterial species in the gut microbiota, and thus covers all three major functional families of bacterial environmental sensors. By investigating the binding specificities of these LBDs for over 150 metabolites known to be present in the mammalian gut, we identified 34 interactions of LBDs with different ligands, including short-chain fatty acids (SCFAs), lactate, formate, indole, pyrimidines, amines, purines, and amino acids. The ability of selected ligands to elicit signaling responses was confirmed by constructing chimeric receptors that could be studied in the heterologous *Escherichia coli* system. Structural analysis and mutagenesis were used to characterize the ligand binding sites for the two prominent groups of domains, providing insights into the evolutionary modification of their ligand binding properties. Most of the identified interactions appear to reflect chemotactic responses to metabolically relevant compounds, highlighting the physiological importance of chemotaxis to nutrients in the gut microbiota. Overall, this study provides an important step toward the functional annotation of bacterial sensors from nonmodel organisms within complex microbial ecosystems.

## Results

### Construction of the LBD Library and High-Throughput Ligand Screening.

We selected an LBD library containing over one hundred extracytoplasmic sensory domains from 20 different human gut bacteria, using our previously established dataset of sensory domains for the human gut microbiome (21) (*SI Appendix*, Table S1 and Dataset S1). The majority of the selected organisms belong to Clostridia, a highly represented and ecologically important class of bacteria in the human gut microbiome, which includes the most abundant motile commensal bacteria. These species thus harbor all three major families of bacterial environmental sensors, with the majority of LBDs in our library derived from MCPs, closely followed by HKs, and DGCs representing the smallest proportion (*SI Appendix*, Fig. S1*A*). Our LBD library consists of three structural groups of domains, including dCache (dual or bimodular) and sCache (single or monomodular) domains of the Cache superfamily and 4HB (four-helix bundle) domains (*SI Appendix*, Fig. S1*B*), which is similar to the distribution of these groups in the original dataset (21). Also consistent with the original dataset, the distribution of domain groups differs between sensor families, with 4HB domains occurring more frequently in MCPs than in HKs (*SI Appendix*, Fig. S1 *C* and *D*).

**Fig. 1.**
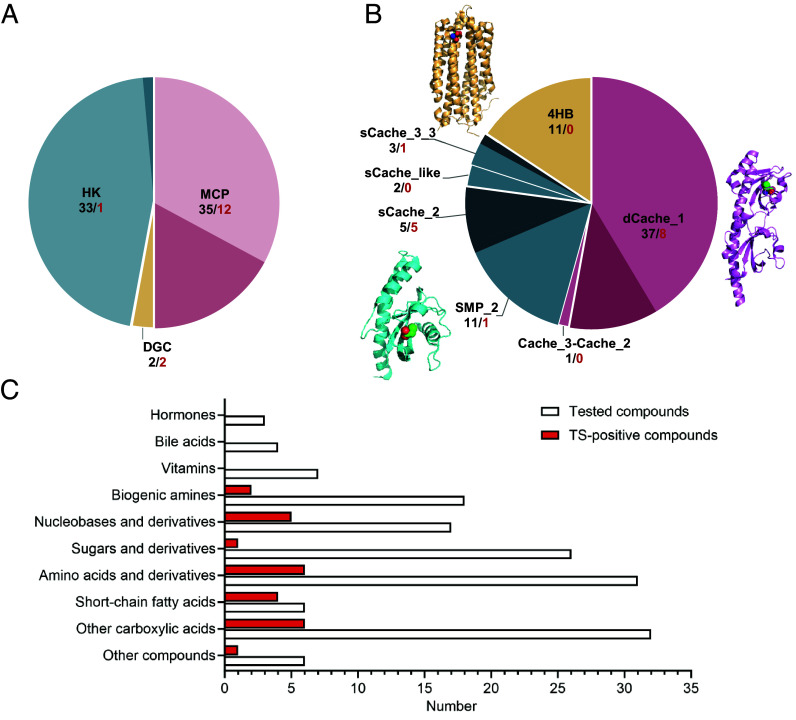
Overview of the high-throughput ligand screening for the LBD and ligand libraries. (*A* and *B*) Composition of the folded LBDs presented by the receptor type (*A*) and by the domain family (*B*), respectively. The numbers below receptor and domain names indicate studied LBDs (black) and those with identified ligands (red). Double-module Cache domain family (dCache) is depicted in pink, single-module Cache domain family (sCache) is represented in blue, and the four-helix bundle_MCP family (4HB) is illustrated in yellow. Fractions of LBDs with identified ligands within different groups are highlighted in darker color. Domain definitions follow the Pfam domain nomenclature. MCP, methyl-accepting chemotaxis protein; HK, histidine kinase; DGC, diguanylate cyclase. Three-dimensional structures of three structural domain families are shown in *B*: 4HB—Tar bound to L-aspartate (PDB ID 4Z9H), dCache—PctA bound to L-isoleucine (PDB ID 5T65), and sCache—PscD bound to propionate (PDB ID 5G4Z). (*C*) Distribution between chemical classes for all studied compounds from two human gut metabolite (HGMT) plates and compounds identified as ligands of one or multiple LBDs using TSAs (referred to as TS-positive compounds). Layouts of two HGMT plates are shown in *SI Appendix*, Table S2.

Next, we expressed and purified LBD proteins from this library and subjected them to high-throughput ligand screening using a thermal shift assay (TSA) (23). Most of the purified LBDs exhibited distinct melting curves with increasing temperature (*SI Appendix*, Fig. S1*E*), confirming that these proteins were initially well folded. Their specificities could therefore be characterized based on changes in thermal stability upon ligand binding. The resulting set of 70 purified and folded LBDs had a similar representation of the receptor families as the original library (Fig. 1*A* and Dataset S1 and *SI Appendix*, Fig. S1 *A* and *F*), despite some enrichment of the Cache domains (Fig. 1*B* and *SI Appendix*, Fig. S1 *B* and *G*.

To screen ligands for these LBDs, we used the custom-assembled HGMT plates containing over 150 different chemical compounds, mainly nutrients, but also several animal hormones and other compounds known to be present in the human gut (Fig. 1*C* and *SI Appendix*, Table S2). We observed binding of one or several compounds to multiple LBDs, including most prominently SCFAs and other carboxylic acids, as well as amino acids and nucleobases and their derivatives (Fig. 1*C* and *SI Appendix*, Table S3 and Fig. S2 *A* and *B*). Interestingly, while we successfully identified ligands for a large fraction of the dCache and sCache domains tested, none of the proteins in the 4HB group exhibited thermal shifts upon addition of any of the compounds tested (Fig. 1*B*). Nevertheless, our TSA setup can be used to characterize ligand binding to the 4HB LBDs, as confirmed for the LBDs of *E. coli* chemoreceptors Tar and Tsr (*SI Appendix*, Fig. S2 *C* and *D*).

In total, we were able to assign ligands for 15 LBDs from nine gut-resident bacterial species, including several *Roseburia* species (*Roseburia inulinivorans*, *Roseburia intestinalis* L1-82, and *Roseburia faecis*), *Hungatella hathewayi*, and *Lachnospira pectinoschiza* (*SI Appendix*, Table S3). Interestingly, despite having a similar number of well-folded LBDs, HKs and MCPs showed a striking difference in their ligand binding properties. While 12 MCP LBDs were able to bind diverse chemical compounds from the HGMT plates, only one HK LBD could be assigned a ligand, indole (Fig. 1*A*). In addition, nucleobases and their derivatives could be assigned as ligands to both tested DGC LBDs.

### Lactate Is a Prevalent Ligand for sCache_2 Domains in the Human Gut Microbiota.

**Fig. 2.**
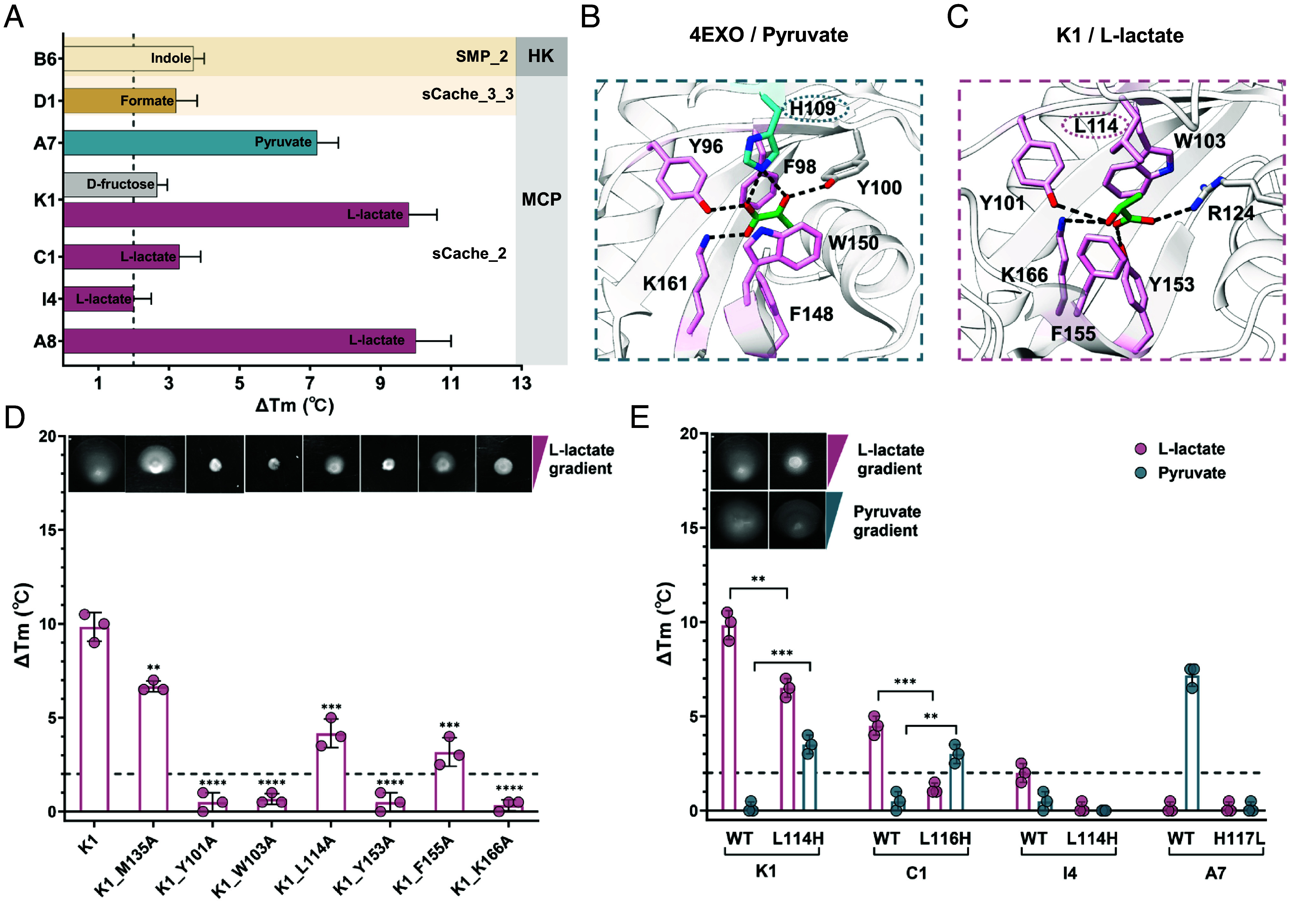
Characterization of the sCache_2 domains specific for short-chain carboxylic acids (SCCAs). (*A*) Thermal shifts observed for sCache domains upon exposure to 2 mM final concentrations of respective ligands. Subfamilies of domains and types of receptors are indicated. The gray dashed line represents the 2 °C threshold used as the significance cutoff for ligand identification. Data are presented as the mean ± SD from three independent biological replicates. (*B*) Reported cocrystal structure of the sCache_2 domain in complex with pyruvate (PDB ID 4EXO). (*C*) Computational docking of L-lactate to the AlphaFold 3 model of K1 LBD. The conformation with the highest confidence is presented. Residues involved in ligand binding are shown in stick mode. The key residues shown in pink indicate conserved amino acids, while variable residues are circled with dashed lines, differentiated by color: blue for H109 in 4EXO and pink for L114 in K1. Black dashed lines present hydrogen bonds. (*D*) Binding and chemotaxis measurements of K1 LBD and its indicated alanine-substituted mutants in response to L-lactate. M135A substitution was used as a negative control. (*E*) Binding and chemotaxis measurements of the indicated LBDs. The chemotaxis measurements in (*D* and *E*) were performed on soft-agar plates with gradients of either L-lactate or pyruvate, indicated by pink or blue triangles. The images shown are representative of three biological replicates. Thermal shifts induced by 2 mM L-lactate are shown in pink, and those by 2 mM pyruvate are shown in blue. The gray dashed line indicates the threshold of 2 °C used as a significance cutoff for ligand identification. The data represent the mean ± SD of three independent biological replicates. Each data point corresponds to an independent biological measurement. Asterisks denote statistically significant differences (unpaired *t* test): **P ≤ 0.01, ***P ≤ 0.001, and ****P ≤ 0.0001.

We assigned several compounds as specific ligands for seven sCache-type LBDs, including L-lactate or pyruvate for five sCache_2 MCP domains, formate for one sCache_3_3 MCP domain containing the recently reported formate binding residues (24), and indole for the SMP_2 HK domain (Fig. 2*A* and *SI Appendix*, Table S3). The binding of L-lactate to the K1 and C1 LBDs (*SI Appendix*, Fig. S3*A*) and the binding of indole to the B6 LBD were confirmed by isothermal titration calorimetry (ITC) (*SI Appendix*, Fig. S3*B*). The ability of the K1 LBD to mediate chemotactic signaling upon L-lactate binding was further confirmed using the K1-Tar chimera, in which this LBD was fused to the signaling domain of Tar. When expressed in the chemoreceptor-less *E. coli* strain, K1-Tar indeed mediated chemotactic responses to L-lactate as determined by the Förster resonance energy transfer (FRET) pathway activity assay (25, 26) (*SI Appendix*, Fig. S4*A*). No response was observed for the same background strain expressing the native Tar receptor (*SI Appendix*, Fig. S4*D*), confirming the specificity of the K1-mediated response to L-lactate.

Since all characterized sCache_2 MCP domains exhibited specificity for L-lactate or pyruvate, the human gut microbiome appears to be enriched in sCache_2 domains dedicated to C2/3 carboxylic acid recognition. To better understand what determines their specificity for lactate or pyruvate, we first identified the structure of a K1-homologous sCache_2 domain from *Vibrio parahaemolyticus* bound to pyruvate (PDB ID 4EXO) in the RCSB Protein Data Bank (PDB) (27) (Fig. 2*B*). Comparison with the molecular docking of L-lactate to the AlphaFold 3 (28) model of K1 LBD (Fig. 2*C*) revealed that both ligands share the same binding mode, with two aromatic amino acids (4EXO^F98^/K1^W103^ and 4EXO^W150^/K1^F155^) sandwiching the ligand and three residues (4EXO^Y96^/K1^Y101^, 4EXO^F148^/K1^Y153^, and 4EXO^K161^/K1^K166^) forming hydrogen bonds with its carboxyl oxygen atoms. These residues are indeed highly conserved in all sCache_2 domains that were shown to bind short-chain carboxylic acids (SCCAs), either in the literature or in our screen (*SI Appendix*, Fig. S5). However, the histidine residue (H109), which also establishes a direct contact with pyruvate in 4EXO and in the pyruvate-binding sCache_2 domains PscD and Adeh_3718 (29, 30), is replaced in all identified L-lactate sensors by leucine (L114 in K1; *SI Appendix*, Fig. S5), which does not directly contribute to binding in the structural model of the K1 LBD complex with L-lactate (Fig. 2*C*). To verify the role of the corresponding amino acid residues in L-lactate binding, we replaced them individually with alanine. Substitutions at any of the four highly conserved residues (Y101, W103, Y153, and K166; numbered according to K1) completely abolished L-lactate binding as measured by TSA, whereas substitutions at F155 and L114 strongly reduced binding (Fig. 2*D*). A lesser effect was observed for the substitution at M135, which is not conserved among these sensory domains. These in vitro binding results were validated by chemotaxis assays for the corresponding alanine-substituted mutants of the K1-Tar chimeric receptor using soft-agar gradient plates (26) (Fig. 2 *D*, *Inset*).

Since the presence of L114 instead of histidine in the binding pocket appears to correlate with the binding of L-lactate, we hypothesized that this difference might be important for ligand selectivity. Indeed, replacing leucine with histidine at this position in the K1 and C1 LBDs enabled the binding of pyruvate but weakened the binding of L-lactate (Fig. 2*E* and *SI Appendix*, Fig. S6). This observation was further confirmed by the chemotaxis assay for the K1-Tar_L114H mutant (Fig. 2 *E*, *Inset*). Thus, we identified L114 in the ligand-binding pocket of sCache_2 domains as the specificity determinant for L-lactate binding.

Although L-lactate produced by host epithelial cells is the predominant lactate enantiomer in the GI tract, intestinal bacteria can produce both L- and D-lactate (31). Therefore, we also examined the binding of D-lactate to the K1 LBD. D-lactate elicited a significant but weaker thermal shift in the K1 LBD compared to L-lactate (*SI Appendix*, Fig. S7*A*) and only a weak response in the FRET assay (*SI Appendix*, Fig. S7*B*). Consistently, the K1 LBD exhibited a four-fold lower binding affinity for D-lactate than for L-lactate (*SI Appendix*, Figs. S3*A* and S7*C*), suggesting that L-lactate is the preferred ligand of this subset of sCache_2 domains.

### Diverse Ligands Bind to the Membrane-Distal Module of dCache_1 Domains.

**Fig. 3.**
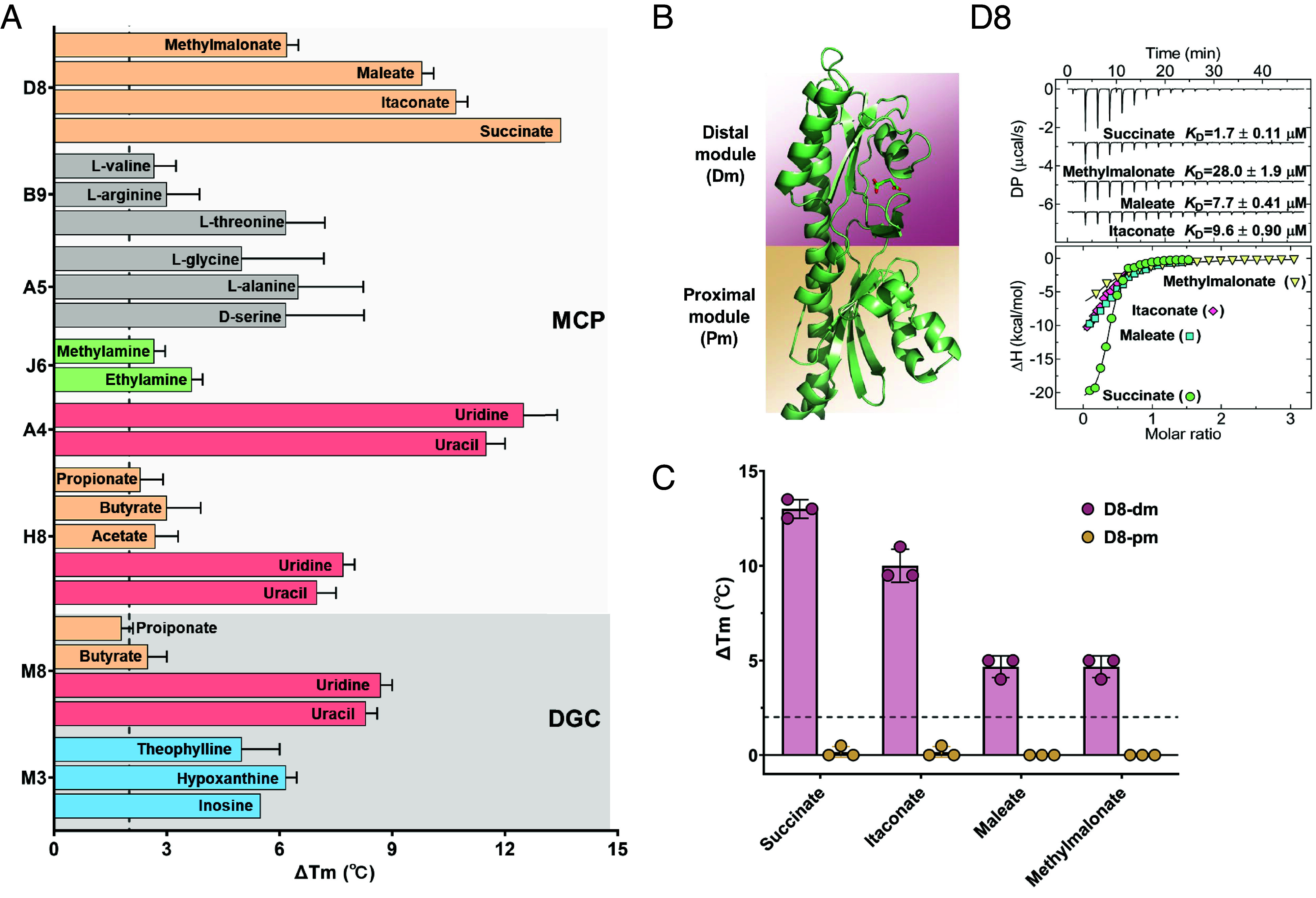
Characterization of diverse ligands binding to the membrane-distal module of dCache_1 domains. (*A*) Thermal shifts observed for dCache_1 domains upon exposure to 2 mM concentrations of respective ligands, with subfamilies of domains and types of receptors being indicated. The gray dashed line represents the 2 °C threshold used as the significance cutoff for ligand identification. Data are presented as the mean ± SD from three independent biological replicates. (*B*) ITC measurements of the D8 LBD binding to indicated ligands, with the derived dissociation constants (*K*_D_) being indicated. *Upper* panel: Raw titration data. *Lower* panel: Integrated, dilution heat corrected, and concentration normalized raw data. Lines correspond to the best fits using the “One binding site” model. Further experimental details are provided in *SI Appendix*, Table S6. The AlphaFold 3 model of the D8 LBD in complex with docked succinate is shown on the left, with the membrane-distal module (dm) colored pink and the membrane-proximal module (pm) colored yellow. (*C*) Thermal shift measurements for the membrane-distal module of the D8 LBD (D8-dm; corresponding to amino acid residues 33–179) and the membrane-proximal module of the D8 LBD (D8-pm; corresponding to amino acid residues 153–280) in the presence of 2 mM concentrations of indicated compounds. The gray dashed line indicates the threshold of 2 °C used as a significance cutoff for ligand identification. The data represent the mean ± SD of three biologically independent replicates. Each data point corresponds to an independent biological measurement.

The spectrum of ligands identified for the dCache_1 domains showed greater diversity, including amino acids, purines, pyrimidines, amines, and carboxylic acids (Fig. 3*A* and *SI Appendix*, Table S3). The membrane-distal modules of the MCP amino acid sensors A5 and B9, the MCP amine sensor J6, and the DGC purine sensor M3 were found to possess recently characterized specificity motifs within their putative binding pockets (32–34) (*SI Appendix*, Fig. S8). The binding of L-threonine to the B9 LBD and of ethylamine and methylamine to the J6 LBD was confirmed by ITC (*SI Appendix*, Fig. S9). Furthermore, the J6-Tar chimeric receptor mediated specific responses to ethylamine and methylamine when measured by FRET in *E. coli* (*SI Appendix*, Fig. S4 *B* and *C*), whereas no response was observed for native Tar used as a control *SI Appendix*, Fig. S4 *E* and *F*.

TSA also revealed binding of the D8 LBD to four C3/C4 dicarboxylic acids—methylmalonate, maleate, itaconate, and succinate (Fig. 3*A*). This interaction was further confirmed by ITC (Fig. 3*B*). To determine which module of the D8 LBD binds these ligands, we separately tested binding to the membrane-distal module (D8-dm) and the membrane-proximal module (D8-pm). Thermal stabilization by all four ligands was observed for the membrane-distal module (Fig. 3*C*), suggesting that binding of C3/C4 dicarboxylic acids occurs at this part of the dCache_1 domain.

### Uracil and SCFAs Independently Bind to Different Modules of dCache_1UR.

Another previously uncharacterized subset of dCache_1 domains, represented by the H8 and A4 MCP sensors and the M8 DGC sensor, was observed to bind two very different classes of compounds: uracil and its derivative uridine, which showed larger thermal shifts and higher binding affinities, and SCFAs, which showed smaller thermal shifts and lower binding affinities (Fig. 3*A* and *SI Appendix,* Fig. S10 *A*–*E*). We therefore named this subset of dCache_1 domains “dCache_1UR.” The H8 LBD was also able to bind another uracil derivative, the anticancer agent 5-fluorouracil (35) (*SI Appendix,* Fig. S10*B*). Given the difference between the molecular structures of SCFAs and uracil, we reasoned that these ligands must bind at different sites. Indeed, the membrane-distal modules of the dCache_1UR domains were able to bind uracil but not SCFAs (*SI Appendix,* Fig. S10 *F* and *G*), suggesting that SCFA binding may instead occur at the membrane-proximal module.

**Fig. 4.**
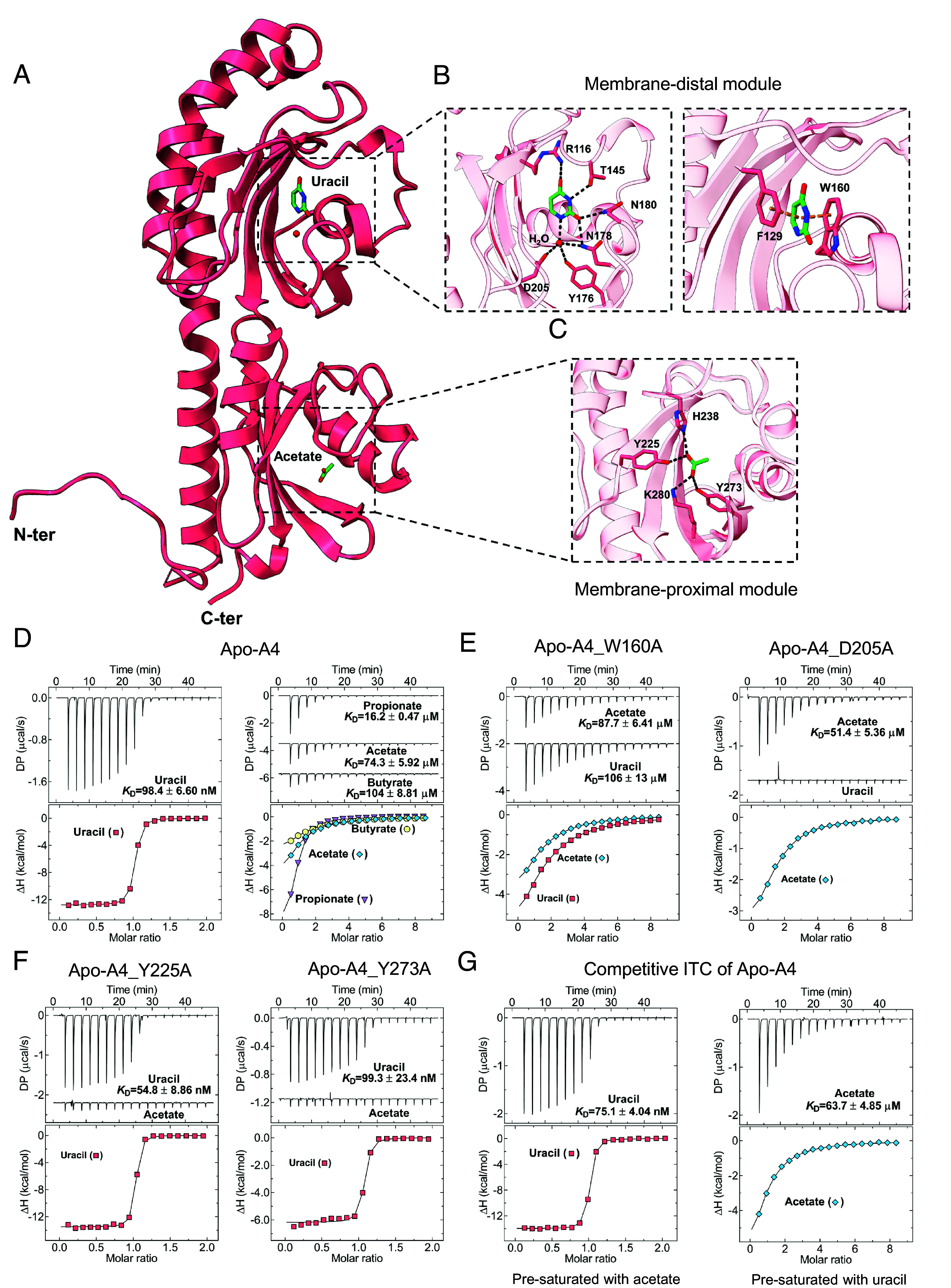
dCache_1UR domain independently binds uracil and SCFAs at distinct modules. (*A*) Overall structure of the dCache_1UR domain A4 LBD in complex with uracil and acetate (PDB ID 9HVJ). (*B* and *C*) Molecular details of uracil recognition sites at the membrane-distal module (*B*) by hydrogen bonds (black dashed lines, *Left*) and π–π stacking interactions (orange dashed lines, *Right*), and acetate recognition site at the membrane-proximal module (*C*) by hydrogen bonds (black dash lines). Residues involved in ligand binding are shown in stick mode. (*D*) ITC measurements of Apo-A4 LBD binding to uracil and SCFAs. (*E* and *F*) ITC measurements for indicated variants of Apo-A4 with amino acid substitutions in the membrane-distal (*E*) or membrane-proximal module (*F*) with indicated ligands. (*G*) Competitive ITC measurements of uracil binding to Apo-A4 presaturated with acetate (*Left*) and of acetate binding to Apo-A4 presaturated with uracil (*Right*). *Upper* panels: Raw titration data. *Lower* panels: Integrated, dilution heat corrected, and concentration normalized raw data. Lines correspond to the best fits using the “One binding site” model, with the derived dissociation constants (*K*_D_) being indicated. Further experimental details are provided in *SI Appendix*, *Materials and Methods* and *SI Appendix*, Table S6.

To elucidate the molecular basis of ligand recognition by the dCache_1UR domains, we crystallized the purified A4 LBD in the presence of uracil and propionate. The resulting structure was determined at a resolution of 1.46 Å (Fig. 4 *A*–*C*). Similar to other dCache_1 domains (14, 15), the A4 domain comprises two modules: the membrane-distal module (residues 57–206) containing seven β-strands, four α-helices, and half of the α1 helix that connects these two modules, and the membrane-proximal module (residues 35–56 and 207–303) formed by four β-strands, five α-helices, and the other half of the aforementioned α1 helix (*SI Appendix*, Fig. S11*A*). Within the membrane-distal module, an electron density that could be unambiguously assigned to uracil was observed (*SI Appendix*, Fig. S11*B*). Uracil is tightly coordinated by eight key residues and one water molecule (Fig. 4*B* and *SI Appendix*, Fig. S11*B*). The residues R116, T145, N178, and N180 stabilize uracil via hydrogen bonds at a distance of ~2.9 Å. Additionally, a water molecule forms hydrogen bonds in a tetrahedral coordination with uracil and residues Y176, N178, and D205, with respective distances of 2.8 Å, 3.1 Å, 2.9 Å, and 2.7 Å (Fig. 4*B*). Moreover, F129 and W160 form π–π stacking interactions with uracil at distances of 3.8 Å and 3.6 Å (Fig. 4*B*). In the proximal module, although propionate was added during crystallization and acetate was not present in the experimental conditions, an acetate molecule was unambiguously explaining the additional density obtained (*SI Appendix*, Fig. S11*C*). Therefore, this acetate molecule was likely endogenous and prebound to the A4 domain during its expression in *E. coli*. Four residues coordinate acetate via hydrogen bonds: Y225, H238, Y273, and K280, with bond distances of 2.6 Å, 2.7 Å, 2.5 Å, and 2.9 Å, respectively (Fig. 4*C*). Consistently, the AlphaFold 3 models of the M8 and H8 LBDs closely resemble the structure of the A4 LBD, with key residues highly conserved (*SI Appendix*, Fig. S10 *H*–*L*).

Further analysis of the purified A4 LBD also revealed the presence of endogenously bound uracil (*SI Appendix*, Fig. S12*A*), suggesting that both binding pockets of A4 are at least partially occupied. To eliminate the likely effects of partial occupancy on binding measurements, prebound ligands were removed from the purified protein to yield a ligand-free A4 LBD (apo-A4; *SI Appendix*, Fig. S12*A*). Indeed, the apo-A4 LBD exhibited much higher affinity (Fig. 4*D* and *SI Appendix*, Fig. S10*A*) and larger thermal shifts to uracil compared to the partially occupied A4 LBD (*SI Appendix*, Fig. S12 *B* and *C*).

To investigate the contribution of individual amino acid residues in the pocket to uracil binding, we tested the effects of alanine substitutions at these residues. Replacement of R116, F129, W160, and D205 residues resulted in a greatly reduced thermal shift (*SI Appendix*, Fig. S12 *B* and *C*) and greatly reduced binding affinity or even complete loss of binding (Fig. 4*E*), whereas replacement of the other four residues had a more modest effect. Similarly, alanine substitution mutants of four key residues in the SCFA binding pocket disrupted binding (Fig. 4*F* and *SI Appendix*, Fig. S12 *B* and *C*).

Next, we investigated whether there might be an allosteric regulation between the binding of SCFAs and uracil to the dCache_1UR domain, using competitive ITC (*SI Appendix*, *Materials and Methods*). No apparent impact of the presence of acetate on the binding of uracil to the A4 LBD or vice versa was observed, suggesting that these ligands bind independently (Fig. 4*G*). Consistent with this, the introduction of single point mutations in the binding pocket for one ligand had no impact on the binding of the other ligand (Fig. 4 *E* and *F* and *SI Appendix*, Fig. S12 *B* and *C*).

### Evolutionary Relatedness between dCache_1UR and Other Characterized dCache_1 Domains.

**Fig. 5.**
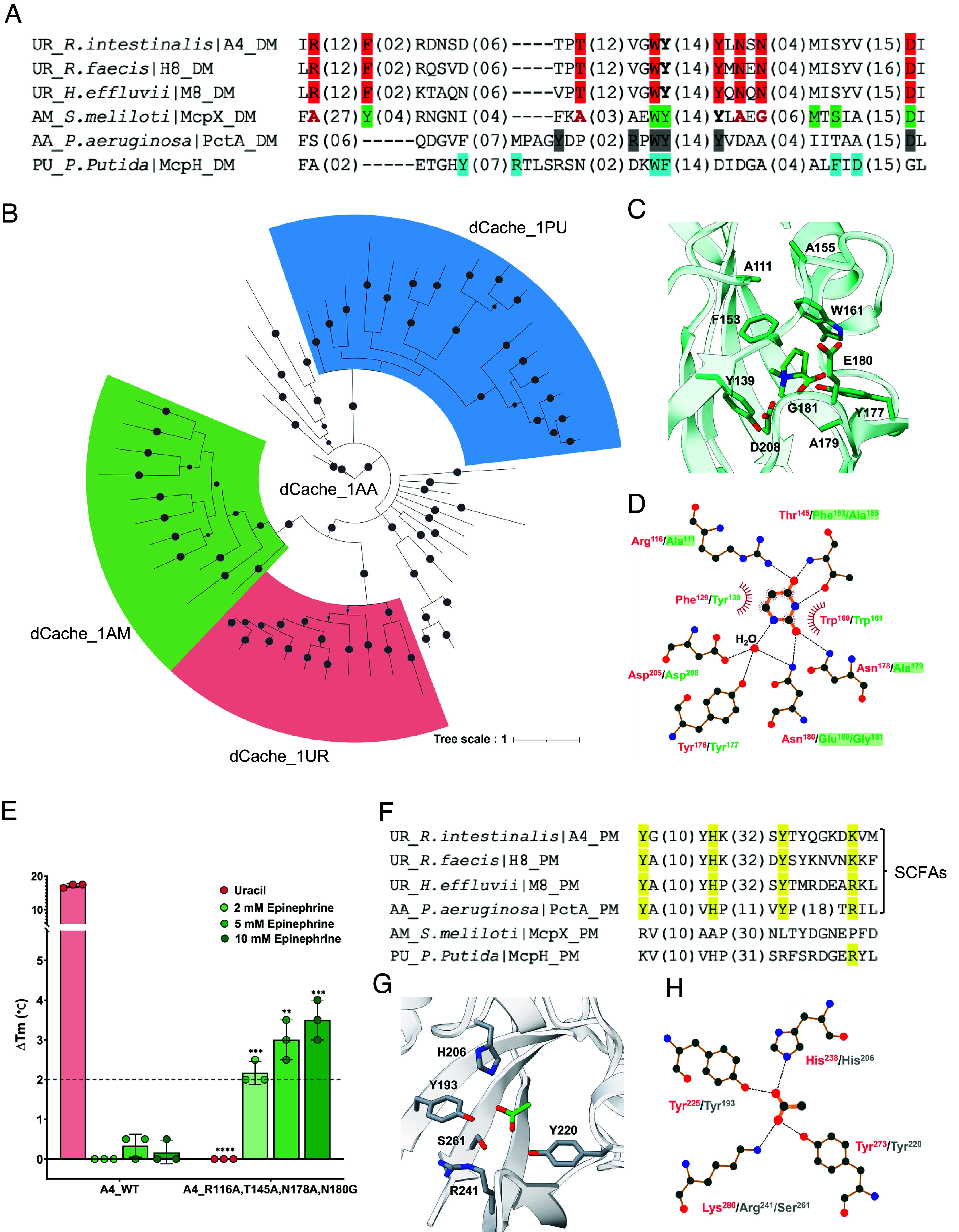
Evolutionary relationships and ligand-binding specificity of dCache_1 domains. (*A*) Multiple sequence alignment of the membrane-distal modules from representative dCache_1 domains known to recognize uracil, amines, amino acids, or purines. The corresponding motifs are shown in red (uracil-binding key residues), green (amine-binding key residues), gray (amino acid-binding key residues), and blue (purine-binding key residues). The four residues of McpX, shown in bold red, are in positions corresponding to the uracil-binding key residues. (*B*) Phylogenetic analysis of the indicated dCache_1 domains. Uracil sensors are marked in red, amine sensors in green, and purine sensors in blue; amino acid sensors are not colored. Probabilities greater than 0.8 are represented by filled gray circles. Amino acid sequences from major phyla of each shown family were used. A detailed phylogenetic tree, including information about the proteins used and their corresponding phyla, is provided in *SI Appendix*, Fig. S13. (*C*) Amine binding pocket of McpX (PDB ID 6D8V) in complex with proline betaine (PBE). (*D*) Schematic representation of residues involved in uracil binding. The residues coordinating uracil in A4 are shown in red, while the corresponding residues in McpX are depicted in green. Residues required for uracil binding but not conserved in McpX are highlighted with a green shadow. Dashed lines represent hydrogen bonds, and half‐circles denote hydrophobic interactions. This figure was generated using LigPlot^+^. (*E*) Thermal shifts observed for the A4 LBD and the A4 _R116A, T145A, N178A, N180G LBD mutant exposed to indicated concentrations of uracil or epinephrine. The data represent the mean ± SD of three biologically independent replicates. Each data point corresponds to an independent biological measurement. (*F*) Multiple sequence alignment of the membrane-proximal modules from uracil, amine, amino acid, and purine sensors. Amino acids involved in SCFA binding are shown in yellow. (*G*) Acetate binding pocket of the amino acid sensor PctA (PDB ID 5T65) in complex with acetate. (*H*) Schematic representation of residues involved in acetate binding. The residues that coordinate the acetate are shown in red (A4) and gray (PctA). Dashed lines represent hydrogen bonds. This figure was generated using LigPlot^+^.

To explore the evolutionary origin of uracil sensors, we performed the sequence alignment for dCache_1UR domains (A4, H8, and M8) with the previously characterized dCache_1AM (McpX), dCache_1AA (PctA), and dCache_1PU (McpH) domains (Fig. 5*A*). It was suggested that dCache_1UR domains shared highest similarity to the dCache_1AM domains. To further elucidate the phylogenetic relationships between the dCache_1UR domain subset and the other three dCache_1 domain subsets, we analyzed corresponding protein sequences representing different taxonomic groups with variations in their motifs (Dataset S2). Phylogenetic inference (Fig. 5*B* and *SI Appendix*, Fig. S13), performed using a Bayesian approach (*SI Appendix*, *Materials and Methods*), suggested that the dCache_1AA domain subset represents the common ancestor of the other three subsets of dCache_1 domains. The dCache_1UR and dCache_1AM domain families form two closely related clades that likely evolved from a shared ancestor. In contrast, the dCache_1PU domains form a separate, well-defined branch. This is consistent with previous analyses indicating that the dCache_1AA domains represent an ancient, broadly distributed domain family (34), with amino acid substitutions and structural adaptations in its distal pocket likely driving the diversification and proliferation of dCache_1 domain families with different ligand specificities.

Consistent with their close relatedness, structural superposition of the ligand-binding pockets of McpX and A4 revealed that four conserved residues (A4^F129^/McpX^Y139^, A4^W160^/McpX^W161^, A4^Y176^/McpX^Y177^, and A4^D205^/McpX^D208^) occupy similar positions and orientations (Fig. 5 *C* and *D*). However, residues R116, T145, N178, and N180, which are also important for uracil binding, show no conservation in McpX, suggesting that these residues may confer specificity for uracil binding (Fig. 5 *A*, *C*, and *D* and *SI Appendix*, Fig. S14*A*). Indeed, replacing these four residues with those from McpX abolished uracil binding and enabled binding of epinephrine, but not other compounds from our HGMT ligand library (Fig. 5*E* and *SI Appendix*, Fig. S14 *B*–*E*).

Furthermore, comparison of the membrane-proximal binding modules revealed conservation of all four key residues for acetate binding between dCache_1UR and dCache_1AA (PctA), but not in the dCache_1AM and dCache_1PU domains (Fig. 5*F*). Consistent with this, bound acetate has been reported in the published structure of the membrane-proximal module of PctA, although the physiological significance of this binding remained unclear (14) (Fig. 5 *G* and *H*). Notably, however, sequence analysis of dCache_1UR homologs indicates that the dCache_1UR and SCFA-binding motifs do not always coexist (Dataset S2).

### SCCAs Are Ubiquitous Chemotactic Signals and Valuable Metabolites for Gut Bacteria.

Given that the majority of ligands identified in our screen targeted bacterial chemoreceptors carrying the Cache superfamily domains, we next focused on gut bacteria containing chemoreceptors of this superfamily. According to the MiST4.0 database (36), 12 out of 20 organisms from our initial set contain chemoreceptor genes, and 11 of these possess Cache-type chemoreceptors (Datasets S1 and S3). Collectively, Cache-type chemoreceptors account for approximately one-third of all transmembrane chemotaxis receptors in these organisms (*SI Appendix*, Fig. S15), with the 4HB and MASE-type domains accounting for the remaining two-thirds. Notably, MASE-type domains were not part of this study because they are characterized by multiple transmembrane regions and lack a clearly structured extracytoplasmic sensory domain (37).

**Fig. 6.**
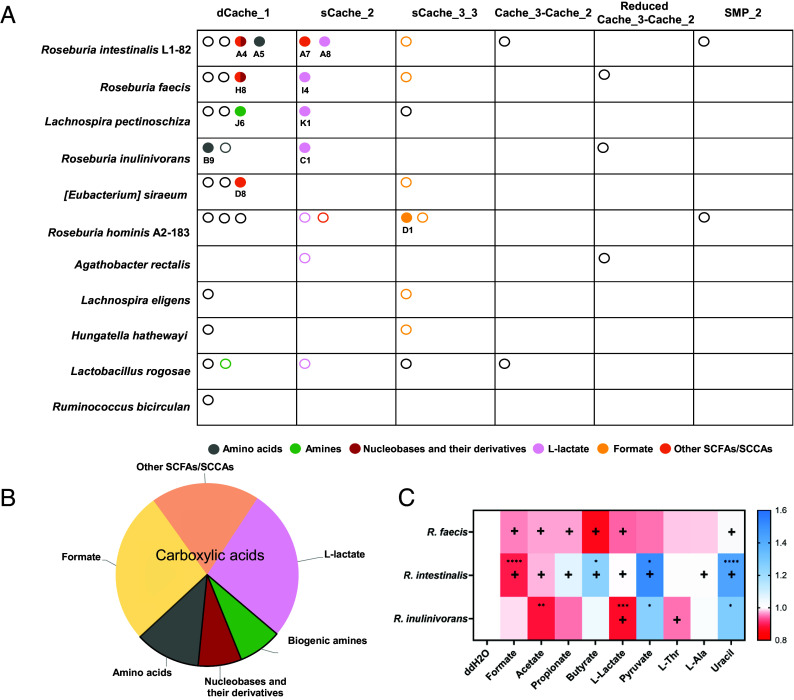
Overview of the chemotactic signals for the Cache superfamily domains of Clostridia in the gut microbiome. (*A*) Chemotactic stimuli profiles of Cache domains in the indicated gut bacteria, identified using the experimental and bioinformatic analyses. Cache domains are categorized based on the domain family. Each LBD is represented by an open circle (predicted stimulus) or a filled circle (experimentally identified stimulus). Colors indicate distinct classes of ligands. (*B*) Composition of identified and predicted chemoeffectors, categorized by classes of chemical compounds, as indicated. L-lactate, formate, and other SCFAs/SCCAs are collectively classified as carboxylic acids. (*C*) Heat map showing the effects of addition of 20 mM of indicated compounds (10 mM for uracil) on growth of the selected *Roseburia* species in 50% YCFA medium (see *SI Appendix*, Fig. S16 for respective growth curves), quantified as the time required to reach the maximal growth rate (t(μ_max)). The symbol “+” indicates the indicated strain possesses the identified or predicted chemoreceptor responsive to the corresponding compound. Data represent mean values from three biological replicates. *P* values (*P ≤ 0.05, **P ≤ 0.01, ***P ≤ 0.001, and ****P ≤ 0.0001) are assessed from a two-tailed unpaired *t* test.

We then integrated the experimentally identified ligand specificities with predictions based on sequence alignment (Fig. 6*A* and *SI Appendix*, Fig. S8). This allowed us to assign ligands to 50% of the Cache family domains, including all sCache_2 domains, most of the sCache3_3 domains, and about a third of the dCache_1 domains (*SI Appendix*, Fig. S15). Among the different classes of metabolites, carboxylic acids were particularly prevalent chemotactic stimuli (Fig. 6*B*). The most common seem to be sensors for L-lactate and formate, which are carried by the majority of the organisms studied, strongly suggesting the physiological importance of locating sources of these two metabolites for the studied gut bacteria. A particularly striking example is *Roseburia hominis*, which is predicted to carry sensors for both L-lactate and pyruvate, as well as two sensors for formate. In contrast, fewer bacteria possess Cache sensors for the commonly studied and more abundant SCFAs (acetate, propionate, and butyrate). Importantly, the *K*_D_ values for different intestinal metabolites are within their known natural concentration ranges (*SI Appendix*, Table S4). Thus, receptor affinities appear to be inversely correlating with the metabolite concentrations in the gut, with the highest affinities for low-abundant metabolites and only low affinities for high-abundant SCFAs.

Such a correlation may be consistent with the presumed primary physiological function of bacterial chemotaxis, namely to locate sources of limiting nutrients and thereby enhance their acquisition (17, 18, 38). Therefore, we directly evaluated the impacts of the major identified chemoeffectors on the growth of three species, *R. intestinalis* L1-82, *R. faecis*, and *R. inulinivorans*. Although no direct correlation was observed between the growth effect of a specific metabolite and the presence of its chemoreceptor, most carboxylic acids, except pyruvate, promoted the growth of at least one of the tested species (Fig. 6*C* and *SI Appendix*, Fig. S16). The most significant impact on growth resulted from supplementation of the medium with formate or L-lactate and was observed for strains that possess respective receptors, supporting the hypothesis that chemotaxis toward carboxylic acids may be due to their nutritional value for gut commensals. In contrast, the effect of uracil supplementation was surprisingly growth inhibitory, and the most significant impact was again observed for *R. intestinalis* L1-82 that possesses the corresponding chemoreceptor.

## Discussion

The gut microbiota is a complex community that relies on the extensive exchange of metabolites and signals among microorganisms and between microbes and the host (39, 40). However, it remains largely unknown which signals are recognized by the diverse extracytoplasmic sensory domains that provide inputs for signal transduction pathways in gut bacteria. Previous systematic efforts to identify ligands of bacterial sensors have focused on single types of sensory domains (32–34, 41) or on several individual model organisms, primarily pathogens (7, 26, 42–44). Here, we instead took a habitat-centric approach and evaluated ligand specificities for a library of 116 LBDs from the recently established dataset of ~17,000 sensory domains of human gut commensal bacteria (21). We focused primarily on the bacterial class of Clostridia, as this class is highly prominent and important in the human gut microbiome (22), and many of its members are potentially motile and therefore possess all three major types of bacterial transmembrane sensors, including MCPs, HKs, and DGCs.

In total, our screening of these LBDs against a library of metabolites known to be present in the gut identified 34 receptor–ligand interactions, revealing distinct patterns of ligand distribution between the functional and structural families of bacterial sensors. Although the LBD library had nearly equal proportions of folded MCP and HK LBDs available for ligand binding assays, nearly all identified ligands were MCP-specific. Ligands could be mapped to 12 out of 35 MCP LBDs, but only to one out of 33 HK LBDs. A possible explanation for this striking difference could be that most of the tested ligands are potential nutrients (41). Bacterial chemotaxis receptors are thought to preferentially recognize nutrients favored by particular bacteria, facilitating foraging and movement toward optimal growth environments (17, 18, 38, 41). Indeed, two recent compilations suggest that approximately three-quarters of all known bacterial chemoattractants serve as growth substrates for the respective bacteria (41, 45).

In contrast, two-component systems often regulate diverse stress responses and may therefore recognize signals molecules other than nutrients, including metal cations or antimicrobial peptides (46, 47), that were not included in our ligand library. Consistent with this distinction between the ligand repertoire of these different functional classes of receptors, less than one-quarter of HKs were reported to respond to nutrients (41). The only ligand identified for a sensory HK from *Catenibacterium mitsuokai* in our screen was indole, which is a ubiquitous bacterial signaling molecule rather than a nutrient (48, 49). Notably, despite the potentially widespread importance of indole as a bacterial signaling molecule in a number of natural environments, including the human GI tract (49, 50), no bacterial sensors with biochemically demonstrated indole binding to their extracytoplasmic sensory domains have been previously described. Another sCache domain from *E. coli* BaeS HK has also been shown to respond to indole, but the underlying mechanism remained unknown (51).

This observed difference in LBD coverage between MCPs and HKs was even more surprising since HKs contained a significantly higher proportion of LBDs from the Cache superfamily, to which all LBDs characterized in our study belonged. The Cache superfamily has been classified into more than a dozen families strictly based on sequence similarity, rather than ligand-binding profiles, with the dCache_1 family being the most abundant and the best-characterized class of bacterial extracytoplasmic sensors (52). In contrast, no ligands were identified for eleven folded LBDs from the 4HB superfamily included in our screen. One possible reason for this could be the insufficient stability of the ligand binding pocket of purified 4HB domains, since they usually bind ligands at the interface between the two monomers (53), despite some exceptions (54). This differs from the LBDs of the Cache superfamily, which have one or two well-defined ligand-binding module(s) within the LBD monomer (41). Our control experiments with the 4HB domains of *E. coli* MCPs and previous studies show that the ligand binding of 4HB domains can be characterized using TSA (26, 55). Alternatively, the lack of identified ligands may indicate that 4HB domains more commonly interact with the ligand-loaded solute binding proteins (SBPs) than with the ligands themselves. Indeed, while all four *E. coli* 4HB domain MCPs are known to interact with SBPs, only two have been shown to bind ligands directly (7). Protein-mediated signaling may also provide an alternative explanation for the lack of identified HK ligands, as at least some of the Cache domains of HKs have been shown to bind SBPs (56) and stress-responsive HKs are also known to sense proteins as signals (47). We further cannot exclude the possibility that in some cases TSA fails to detect ligand binding when it does not significantly alter protein stability. Nevertheless, as mentioned already for the HK LBDs, the most likely explanation for our inability to identify ligands for many well-folded LBDs is that their cognate ligands were not present in our library.

The spectrum of nutrient metabolites identified as MCP ligands in our study shows a high diversity, including amino acids, amines, and pyrimidines. The most prevalent ligands, however, were carboxylic acids, where we identified three different binding modes: 1) sCache_2 domains that bind lactate or pyruvate; 2) a dCache_1 domain that binds dicarboxylates at its membrane-distal module; and 3) dCache_1 domains (named here dCache_1UR) that bind SCFAs at their membrane-proximal and uracil at their membrane-distal modules. The latter example is particularly intriguing because, although dCache domains contain two putative ligand-binding modules (57), only a few examples of ligand binding to both sites within the same sensor have been described experimentally (58–60)_._

Our structural and phylogenetic analysis of the dCache_1UR domain complex with two ligands allows us to draw several conclusions about the evolution of this subset of dCache_1 domains. We demonstrated that the uracil-binding pocket is closely related to that of dCache_1AM (amine) sensors, and its specificity could be altered from uracil to amine by several amino acid substitutions. Remarkably, this alteration yielded a sensor for epinephrine, a human catecholamine hormone. Although its physiological relevance remains to be investigated, epinephrine is known to play an important role in host–microbiota interactions (61), and our results demonstrate that dCache_1 domains can readily evolve specificity for epinephrine. In contrast to the uniqueness of the uracil-binding distal pocket, the SCFA-binding proximal pocket of dCache_1UR shows high similarity to the proximal pocket of the amino acid sensor PctA, which was indeed previously cocrystallized with acetate (14). Our analysis suggests that the specificities of the two pockets are not strictly coupled, but the detailed evolution of these receptor modules requires further investigation. Thus, the sensory domains of the Cache superfamily can readily adapt to recognize novel ligand combinations as bacteria colonize new environmental niches. Further highlighting this evolvability, we show that the specificity of sCache_2 domains can be converted from L-lactate to another carboxylic acid by a single amino acid substitution.

Finally, our results provide general insights into the chemotactic preferences of commensal bacteria in a specific environment, the human gut. Overall, we were able to identify or predict ligand specificities for 50% of the Cache superfamily sensors in the commensal bacteria studied. While the better-studied sensors for amines and amino acids appear to be only sparsely represented, the less-studied sensors for carboxylic acids are highly abundant in these bacteria. This preference strikingly differs from the chemoattractant spectrum of known bacterial species, where the most common attractants are amino acids and peptides, benzenoids, and purines (45), indicating high nutritional value of carboxylic acids in the human gut. Interestingly, the typical ligands within this group are not the commonly studied and highly abundant SCFAs, but rather L-lactate and formate. Despite their lower concentrations, L-lactate and formate are likely to play important roles in the gut. L-lactate is produced in significant amounts by the host (62), likely forming gradients toward the intestinal epithelium. Moreover, both L- and D-lactate, as well as formate, can be excreted by many gut bacteria as products of anaerobic mixed-acid fermentation (62–64), thus likely creating local sources of these metabolites. The observed detection of both lactate enantiomers, but with a preference for L-lactate, suggests that both host- and bacterial-derived lactate may be important signals for gut Clostridia. Both lactate and formate play important roles in bacterial cross-feeding under anaerobic conditions, serving as nutrients and/or electron donors for anaerobic respiration (65–67). These conclusions are consistent with our observation of effects on the growth of selected commensal bacteria, with L-lactate and formate providing the most significant growth enhancement among the chemoeffector metabolites tested. This observation is further supported by the LBD affinities, with the highest affinities observed for the less abundant metabolites. Finally, L-lactate secreted by the host cells may not only serve as a nutrient, but also as a sensory cue that allows bacteria to orient themselves in the GI tract, as has previously been proposed for other host-derived signals (68). Such multiple roles of L-lactate—as a nutrient, signaling molecule, and chemoattractant—have been implicated in infection by the gastric pathogen *Helicobacter pylori* (69, 70). Interestingly, the LBD type of the L-lactate-specific chemoreceptor of *H. pylori*, TlpC, is dCache_1 rather than sCache_2 (70), further illustrating that different types of sensory domains can evolutionary converge on the same ligand.

Interestingly, although DGCs are not usually considered to be nutrient sensors (16), nucleobase derivatives and/or SCFAs were also identified as ligands for both tested sensory domains of DGCs. In the latter case, the DGC LBD belonged to the same dCache_1UR domain family as chemoreceptor sensors, demonstrating its recent evolutionary exchange among gut bacteria. However, the extent to which DGCs in commensal bacteria can sense nutrients remains to be investigated in a broader set of transmembrane DGC sensors.

## Materials and Methods

Bacterial strains, plasmids, and primers used in this study are listed in *SI Appendix*, Table S5.

Construction of chimeric chemoreceptors, chemotaxis assays, and FRET measurements were carried out as previously described (25, 26, 71). Protein expression, purification, TSAs, and ITC measurements were performed using published protocols (21). Detailed description of these and other experimental procedures, including gut bacterial growth measurements, HPLC analyses, and crystallization and structure determination, is provided in *SI Appendix*.

Bioinformatic analyses of protein sequences and structures were performed using tools detailed in *SI Appendix*. Multiple sequence alignments were built using MAFFT (72), computational docking was carried out using DiffDock (73), and phylogenetic tree analysis was performed using MrBayes (74).

## Supplementary Material

Appendix 01 (PDF)

Dataset S01 (XLSX)

Dataset S02 (XLSX)

Dataset S03 (XLSX)

## Data Availability

The structure factors and coordinates have been deposited in the Protein Data Bank (PDB, www.rcsb.org) with the accession codes: 9HVJ (75). All other data are included in the manuscript and/or supporting information.
