# Supplementary material for: Specificities of chemosensory receptors in the human gut microbiota
Source: Proc Natl Acad Sci U S A. Author manuscript; Available in PMC 2025 Sep 16. (PMC12415202; doi:10.1073/pnas.2508950122)
Supplement: Supplementary Material [file NIHMS2107169-supplement-Supplementary_Material.pdf]

PNAS

Supplementary Information

Specificities of Chemosensory Receptors in the Human Gut Microbiota

Wenhao Xu, Ekaterina Jalomo-Khayrova, Vadim M Gumerov, Patricia A. Ross, Tania S. Köbe, Daniel Schindler, Gert Bange, Igor B. Zhulin, Victor Sourjik

*Address correspondence to Victor Sourjik (victor.sourjik@mpi-marburg.mpg.de), Igor Zhulin (jouline.1@osu.edu), or Gert Bange (gert.bange@synmikro.uni-marburg.de)

This PDF file includes:

SI Materials and Methods
Figures S1 to S16
Tables S1 to S7
Legends for Datasets S1 to S3
SI References

Other supporting materials for this manuscript include the following:

Datasets S1 to S3 (separate file)

SI Materials and Methods

Bacterial strains, plasmids, and growth conditions

Bacterial strains, plasmids, and primers used in this study are listed in *SI Appendix*, Table S5. For molecular cloning and protein expression, *E. coli* strains were grown in Luria broth (LB; 1% tryptone, 0.5% yeast extract, and 1% NaCl) at 37 °C with shaking. For chemotaxis and FRET experiments, *E. coli* strains were grown in tryptone broth (TB; 1% tryptone and 0.5% NaCl) at 34 °C with shaking. When necessary, antibiotics were used at the following final concentrations: kanamycin, 50 µg/ml; ampicillin, 100 µg/ml; and chloramphenicol, 34 µg/ml. For the gut bacteria growth assays, detailed information is provided as a separate section.

Construction of protein expression plasmids and chimeric chemoreceptors

The protein sequences of studied extracytoplasmic sensory domains (Dataset S1) were back-translated into DNA sequences using EMBOSS Backtranseq (1). Subsequently DNA sequences were codon-matched to *E. coli* using DNA Chisel (2). During the process, the following enzyme recognition sites were excluded: BsaI, BsmBI, EcoRI, NotI, PstI, SpeI, and XbaI. Synthetic DNAs were ordered as fragments from Twist Bioscience. All protein expression plasmids were generated by Gibson Assembly. In brief, the periplasmic sensory domains with overlapping sequences of vector pET28a (+) were amplified by PCR using the synthetic DNA fragments as templates. The resulting fragments were assembled into the linearized vector pET28a (+) (digested by NdeI and BamHI) using in-house generated Gibson Assembly mix (3). Single amino acid mutations were generated using the Q5 site-directed mutagenesis kit (New England BioLabs) following the manufacturer's instructions. All constructs were verified by external Sanger DNA sequencing services.

To construct chimeric chemoreceptors, the amplified LBD fragments containing the overlapping sequences of vector pKG116 and random linkers were cloned into the linearized vector pKG116 digested by NdeI and BamHI. After cloning, the functional chimeras were selected from a library of the LBD [1-X]-XXXXX-Tar [203-553], which contains a five-amino acid random linker between the LBD and Tar signaling domain, as described previously (4).

Soft-agar chemotaxis assays

The chemotaxis assay was conducted on semi-solid minimal A agar plates (0.25% (w/v) agar, 10 mM KH_2PO_4/K_2HPO_4, 8 mM $(NH4)_2SO_4$, 2 mM citrate, 1 mM $MgSO_4$, 0.1 mg/ml of thiamine-HCl, 1 mM glycerol, and 40 µg/ml of a mixture of threonine, methionine, leucine, and histidine) supplemented with appropriate antibiotics and inducers. After solidification, 200 µl aliquots of 100 mM chemical solutions were applied as a line to the center of the plate and incubated at 4 °C for 16 h to form a chemical gradient. The chemoreceptor-less *E. coli* cells expressing the chimera as a sole chemoreceptor were plated approximately 2.5 cm away from the line where the chemical was applied, and plates were incubated at 30 °C for 24 - 48 h.

To select a functional chimeric chemoreceptor with a random linker, the library was applied to a soft agar plate with D-glucose gradients for three rounds of selection. D-glucose is a nonspecific chemoattractant that can be sensed via the phosphotransferase system (PTS) which transmits signals to the cytoplasmic region of chemoreceptors independently of the sensory domain (5). Strains that migrated furthest in the D-glucose gradient were re-inoculated on a new plate for the next round of selection. After three rounds of selection, chimera expression plasmids for the best-chemotactic cells were isolated and their linkers were identified by external Sanger DNA sequencing services.

FRET measurements

FRET measurements were performed as described previously (6, 7). Chemoreceptor-less *E. coli* strains VS181 with the plasmids encoding chimeric chemoreceptor and CheY-YFP/CheZ-CFP FRET pair were grown in 10 ml TB medium supplemented with appropriate antibiotics and inducers (50 µM IPTG and 1-2 µM sodium salicylate) at 34°C and 275 r.p.m. Cells were then harvested at OD_{600} of 0.5 by centrifugation and washed twice with tethering buffer (10 mM KH_2PO_4/K_2HPO_4, 0.1 mM EDTA, 1 µM methionine, 10 mM sodium lactate, pH 7.0), whereas the responses to lactate were measured using tethering buffer without sodium lactate. For microscopy, the cells were attached to the poly-lysine-coated coverslips for about 10 - 15 min and mounted into a flow chamber that was maintained under a constant flow of 0.3 ml/min of tethering buffer using a syringe pump (Harvard Apparatus) that was also used to add or remove compounds of interest. Given pH is a prevalent chemotactic stimulus, the pH value of all tested compounds was adjusted to 7.0. FRET measurements were performed on an upright fluorescence microscope (Zeiss AxioImager.Z1) equipped with photon counters (Hamamatsu). Finally, the fluorescence signals were recorded and analyzed as described previously (6). D-glucose was routinely used as a nonspecific chemoattractant to assess the activity of hybrid chemoreceptors.

Expression and purification of ligand binding domains

E. coli T7 Express strains (New England BioLabs) carrying the LBD expression plasmids were grown in LB medium supplemented with kanamycin at 37 °C until the optical density at 600 nm (OD_{600}) reached 0.6. Isopropyl-β-D-thiogalactoside (IPTG) was added to induce protein expression at a final concentration of 0.1 mM. Growth was continued at 18 ºC for 12 h and cells were collected by centrifugation. Proteins were purified by metal affinity chromatography using modified procedures for His GraviTrapTM column. Briefly, cell pellets were resuspended in binding buffer (20 mM sodium phosphate, 500 mM NaCl, and 20 mM imidazole, pH 7.4) supplemented with 0.2 µg/ml lysozyme, 1 mM $MgCl_2$, 1 mM PMSF, stirred for 30 min at 4 ºC and disrupted using an ultrasonic homogenizer followed by centrifugation at 20,000 x *g* at 4 ºC for 30 min. Afterward, the supernatant was loaded into His GraviTrap^{TM} column previously equilibrated with binding buffer. Following two washing steps with binding buffer, proteins were eluted by elution buffer (20 mM sodium phosphate, 500 mM NaCl, and 500 mM imidazole, pH 7.4). Finally, the eluted protein fractions were dialyzed against dialysis buffer (10 mM sodium phosphate, 150 mM NaCl, 10% (v/v) glycerol, pH 7.0) and concentrated using Amicon Ultra-15 centrifugal filters. For proteins used in ITC experiments, an additional purification step was performed by size-exclusion chromatography (SEC) on an S200 XK16 column (Cytiva) using a buffer with the same composition as the dialysis buffer.

To remove the bound acetate and uracil from the A4 LBD, the protein was purified under previously established conditions with the addition of two extra washing steps during the affinity purification. Briefly, the cleared lysate was loaded into 1 ml HisTrap HP column (GE Healthcare), followed by three sequential washing steps prior to elution: 1) 20 column volumes (CV) of binding buffer, 2) 10 CV of washing buffer, and 3) 20 CV of binding buffer. To evaluate the best conditions for removing the pre-bound ligands, different washing buffers were tested: 1) 20 mM sodium phosphate, 2 M NaCl, pH 7.4; 2) 20 mM sodium phosphate, 3 M Urea, pH 7.4; 3) 20 mM sodium phosphate, 5 M Urea, pH 7.4; and 4) 20 mM sodium phosphate, 7 M Urea, pH 7.4. The purified protein was analyzed using HPLC to determine the amount of uracil present in the sample. For subsequent experiments, the washing solution containing 5 M urea was selected.

Thermal shift assays

Thermal shift assays were performed in 384 microtiter plates using a Bio-Rad CFX384 Touch^{TM} Real-Time PCR instrument. The tested compounds are derived from two HGMT (human gut metabolites) plates that are preconfigured 96 well plates containing about 150 different chemical compounds, as

demonstrated in *SI Appendix*, Table S2. All compounds were dissolved in H_2O at the final concentration of 20 mM and pH 7.0. Each 25 µl assay mixture consisted of 20.5 µl of purified protein (30 -100 µM), 2 µl of SYPRO™ Orange (Invitrogen) at a 5x concentration, and 2.5 µl of chemical solution from HGMT plates, resulting in 2 mM final concentration of tested ligands, unless otherwise indicated. Samples were gradually heated from 23 °C to 95 °C at a rate of 1 °C per min. The unfolding of proteins was tracked by detecting changes in fluorescence. This process enabled the determination of the midpoint of the protein unfolding transition, also known as the melting temperature (Tm), by utilizing the first derivative values of the raw fluorescence data. Data analysis was conducted using Bio-Rad CFX Manager 3.1 software.

ITC measurements

Ligands and proteins were diluted in a buffer containing 10 mM sodium phosphate, 150 mM NaCl, 10% (v/v) glycerol, pH 7.0. The purified proteins were titrated in the sample cell at a final concentration of 25-91 µM each (*SI Appendix*, Table S6). The protein concentrations were predetermined by absorbance at 280 nm. The ligands were placed in the titration syringe at a nominal concentration of 0.1 to 10 mM to saturate the protein sample during the titrations. All the measurements were performed at 25 °C with the instrument MicroCal PEAQ-ITC (©Malvern Panalytical) with a method consisting of 19 injections (first 0.4 µl, and the rest 2 µl each) and 150s of spacing. The raw data were processed with the MicroCal PEAQ-ITC Analysis Software using the "one binding site" model and plotted using GraphPad Prism v8.4.3.

Competitive ITC measurements

Competitive ITC measurements were performed to investigate the interplay between ligand-binding events mediated by two distinct ligand-binding modules. The detailed protocol was modified from the previous publication (8). Briefly, 66-69 µM of Apo-A4 LBD protein was incubated in the buffer containing 10 mM sodium phosphate, 150 mM NaCl, 10% (v/v) glycerol, pH 7.0, and supplemented with either 700 µM uracil or 3 mM acetate. For titration, the same buffer as for protein preparation was used, containing either 700 µM uracil or 3 mM acetate. The same conditions were used for the measurement and data analysis, as described above.

Protein structure manipulations, computational docking, and protein sequence alignments

The structures of the target proteins (M8, H8, D8, and K1) were built using AlphaFold3(9). The crystal structures of the pyruvate sensor (PDB ID 4EXO), PctA (PDB ID 5T65), and McpX (PDB ID 6D8V) were taken from the RCSB protein data bank (10). Comparative analysis of solved and modeled protein structures was performed using PyMOL 3.0 and/or ChimeraX 1.8 (11). Figures were generated by ChimeraX 1.8. For the *in-silico* docking, ligands were downloaded from PubChem database (12) in SDF format. DiffDock (13) was used to computationally dock ligands to Cache domain models using the default settings. Docking results were viewed and interpreted using ChimeraX 1.8. Protein sequence alignments were built using the L-INS-I algorithm of the MAFFT package (14) with default setting and visualized in Jalview (15) to identify the presence of corresponding ligand-binding motif, employing the previously well-characterized motif containing sensors as a target.

To identify dCache_1UR homologs, two iterations of PSI-BLAST searches were initiated against the RefSeq database (release 226) with a maximum number of target sequences of 20,000 using the dCache_1 domain of the uracil sensor A4. Protein sequence regions corresponding to the dCache_1 domain were extracted, with sequence redundancy reduced at 100% identity, and a multiple sequence alignment was constructed using the MAFFT (v. 7.490) (14) L-INS-I algorithm. Uracil and the putative SCFA motif residues were then identified and tracked on the alignment. Motif variant identification and taxonomy information retrieval were performed using a custom Python script.

Phylogenetic tree analysis

The multiple sequence alignment of dCache_1 protein sequences prepared to build the tree was edited using an alignment trimming tool, trimAl (v. 1.4.1) (16): positions in the alignment with gaps in 10% or more of the sequences were removed unless this leaves less than 60%. In such case, the 60% best (with fewer gaps) positions were preserved. The amino acid replacement model for the set of protein sequences was determined running ProtTest (v. 3.4.2) (17). The best model was found to be LG with gamma distribution of rate variation across sites in combination with the empirical state frequencies (LG + G + F). Using the determined amino acid replacement model, a phylogenetic tree was constructed using a Bayesian inference algorithm implemented in MrBayes (v. 3.2.7a) (18). Metropolis-coupled Markov chain Monte Carlo simulation implemented in MrBayes was run with 3 heated and 1 cold chain, discarding the first 25% of samples from the cold chain at the "burn-in" phase. A total of 2,500,000 generations were run till the sufficient convergence was achieved (the average standard deviation of split frequencies is equal to or less than 0.01; in this case it was 0.008) with chain sampling every 1000 generations.

Gut bacteria growth measurements

Gut bacteria were cultivated at 37 °C under anaerobic conditions in a vinyl anaerobic chamber (COY) inflated with a gas mix of approximately 10% carbon dioxide, 88% nitrogen and 2% hydrogen. Prior to the measurement, frozen glycerol stocks were streaked for single colonies onto YCFA agar plates prepared according to the YCFA medium (ID1611) recipe provided by MediaDive (19), and incubated at 37 °C. A single colony was inoculated in 1 ml YCFA medium and grown at 37 °C with constant shaking.

To monitor the growth effect of chemical compounds, bacteria growth assays were carried out using 50% YCFA medium, which was prepared by diluting the original YCFA medium with an equal volume of distilled water (ddH$_2$O). All test compounds were prepared at a concentration of 20 mM, except for uracil, which was tested at 10 mM due to its poor solubility. Pre-cultures were diluted 1:100 into 500 µl of 50% YCFA medium supplemented with the respective test compound in a 48-well plate. Afterwards, growth was measured as OD$_{600}$ in a plate reader (Infinite M200 Pro, Tecan) at 37 °C with constant shaking at 200 r.p.m. To correct for the medium turbidity, raw growth curves were first normalized with inoculum OD (blank OD). Growth plots were then generated using the GraphPad Prism (v. 10.1.0) and quantification of growth parameters was done using QurvE (20) (non-parametric model). Statistical analyses were performed with GraphPad Prism (v. 10.1.0).

Crystallization and structure determination of the A4 domain

Crystallization was performed using the sitting drop vapor diffusion method at 20 °C in 0.5-0.75 µl drops. The drops consisted of protein and precipitant solutions mixed at 1:1 or 1:2 ratios. The protein solution was prepared by incubating 35 µM of freshly purified His6-A4 with 700 µM uracil and 3 mM propionate for 20 min on ice. Then the protein solution was concentrated to 850 µM using 10 kDa cut-off Amicon Ultra Centrifugal Filter tubes (Millipore). A total of 384 crystallization conditions were screened using the JCSG Core Suite I-IV (Qiagen). Crystals were obtained in a solution containing 0.2 M potassium fluoride supplemented with 20% (w/v) PEG3350. Prior to data collection, crystals were flash-frozen in liquid nitrogen using a cryo-solution consisting of mother liquor supplemented with 20% (v/v) glycerol. Diffraction data were collected under cryogenic conditions at the European Synchrotron Radiation Facility (Grenoble, France) at beamline ID23-2. Data were processed with XDS and scaled with XSCALE (21). The structure was determined by molecular replacement with PHASER (22), using the AlphaFold3 model of A4 LBD. Manual model building was performed in COOT (23), followed by the refinement with PHENIX(24). Figures were prepared using ChimeraX 1.8. Crystallization data collection and refinement statistics are given in *SI Appendix*, Table S7. Structure coordinates and structure factors

of A4 LBD have been deposited in the Protein Data Bank under the accession code (PDB ID): 9HVJ. Topology analysis of the A4 LBD was performed using tools from PDBsum(25) and ChimeraX 1.8.

HPLC analysis

HPLC analysis was conducted using an Agilent 1,100 Series System (Agilent Technologies) equipped with a Metrosep A Supp5 – 150/4.0 column (Metrohm). 10 µl of each sample were injected. Nucleotides were eluted at a flow rate of 0.6 ml/min with 90 mM $(NH_4)_2CO_3$ at pH 9.25 and detected at the wavelength of 260 nm. The sample was prepared as follows: 150 µl of chloroform was added to a 50 µl solution containing 10 µM of purified protein. The mixture was then vigorously agitated for 5 s, heated at 95 °C for 15 s, and immediately snap-frozen in liquid nitrogen. The thaw samples were centrifuged (13,000 r.p.m. for 20 min at 10 °C), and the aqueous phase was transferred to an HPLC vial for analysis. A 50 µl solution containing 1 mM uracil was used as standard and treated using the same procedure as described above. The resulting chromatograms were plotted using GraphPad Prism v8.4.3, and the uracil standard data was transformed using Y=Y/100 to facilitate the visual comparison with sample results.

SI Figures

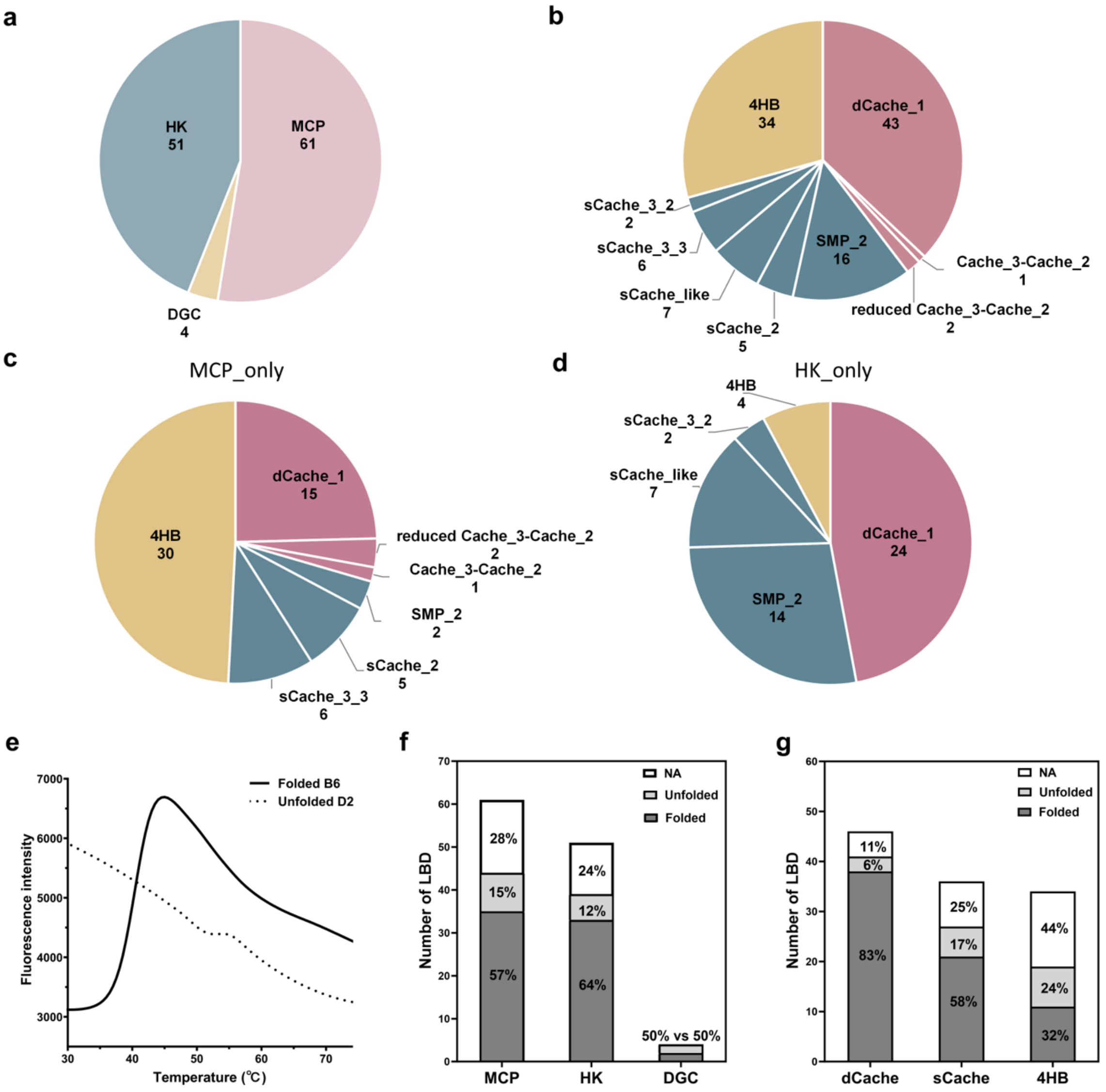

Fig. S1 Functional and structural groups of the synthesized LBDs. **a**, **b**, Composition of the entire LBD library presented by the receptor type (a) and by the domain family (b), respectively. The numbers below each domain or receptor name indicate the number of LBDs in the respective subgroup. **c**, **d**, The proportion of LBDs from different domain families among all chemoreceptors (c) and histidine kinases (d). The underlying data are available in Dataset S1. **e**, Representative melting curves of a folded (B6 LBD) and an unfolded domain (D2 LBD). **f**, **g**, Comparative analysis of protein thermal stability by receptor type (f) and domain family (g). Proteins that could not be expressed or purified, and thus could not be analyzed (NA) by thermal shift assays, are indicated in white; and purified proteins are categorized by thermal stability into unfolded and folded groups, shown in light gray and gray, respectively.

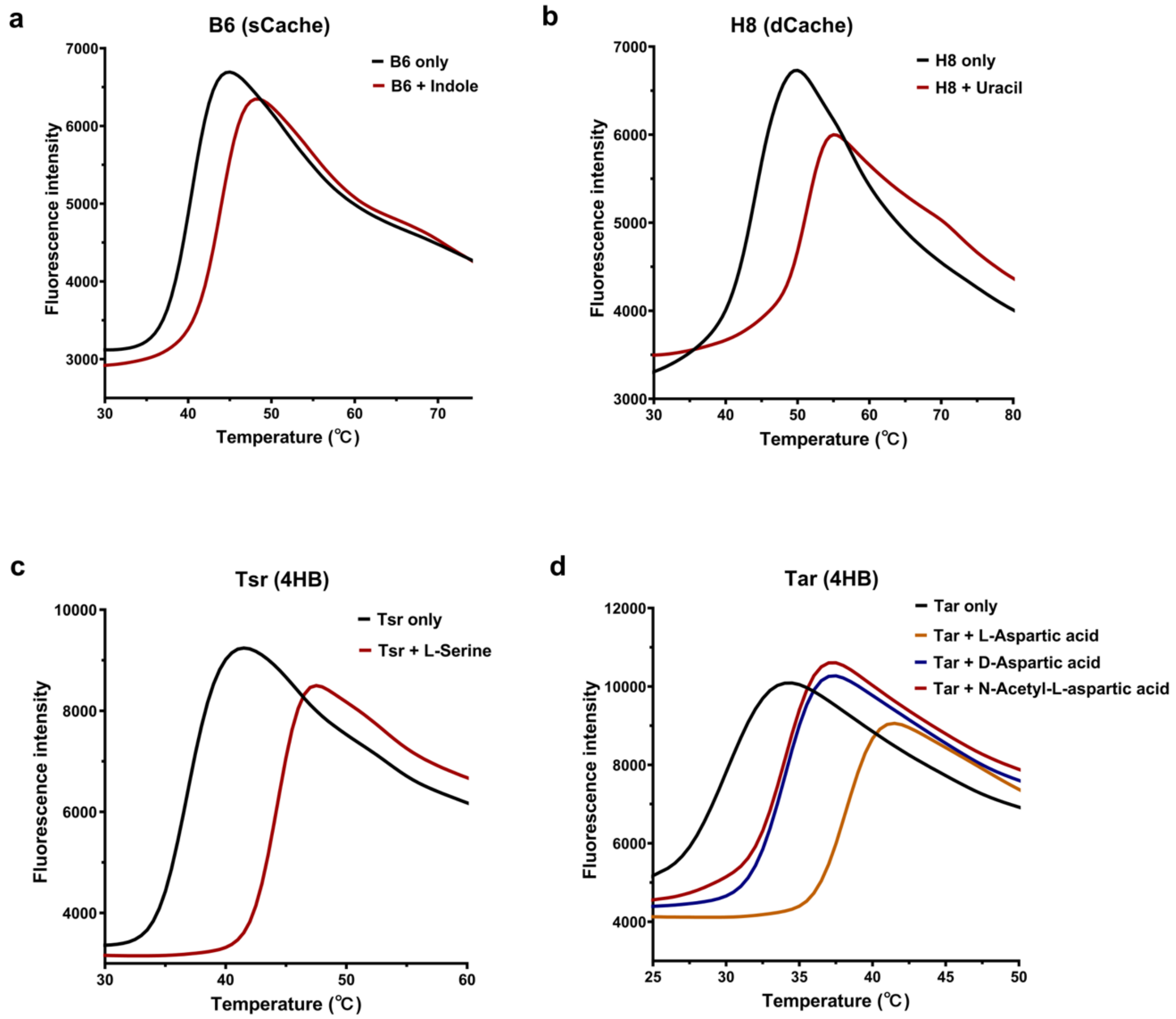

Fig. S2 Melting curves of selected representative sensory domains. a, **b**, Representative thermal shifts of sCache and dCache domains upon addition of 2 mM final concentration of indicated ligands. Thermal unfolding curves of the sCache domain B6 (a) and the dCache domain H8 (b) in the absence and presence of indole and uracil, respectively. **c**, Thermal shift assays of the 4HB-type LBD from *E. coli* Tsr chemoreceptor using two HGMT plates. The Tsr LBD specifically binds L-serine. **d**, Thermal shift assays of the 4HB-type LBD from *E. coli* Tar chemoreceptor using two HGMT plates. The Tar LBD exhibited a positive thermal shift upon stimulation with L-aspartic acid (orange), D-aspartic acid (blue), and N-acetyl-L-aspartic acid (red).

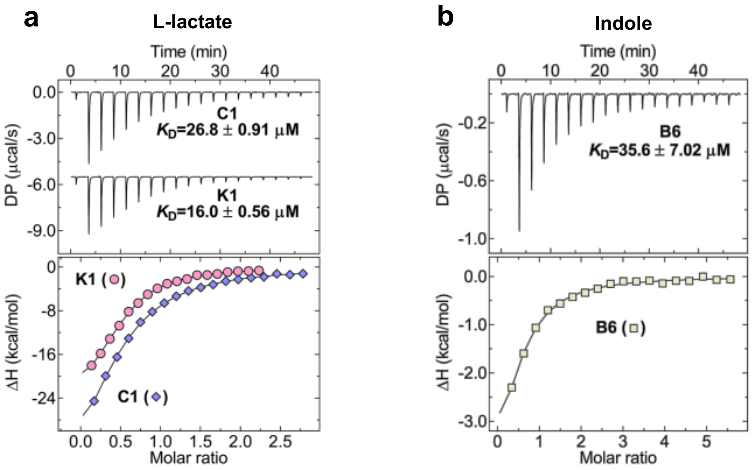

Fig. S3 ITC measurements of ligand binding to sCache domains. a, Binding of the K1 and C1 LBDs to L-lactate. **b**, Binding of the B6 LBD to indole. Upper panels: Raw titration data. Lower panels: Integrated, dilution heat corrected, and concentration normalized raw data. The lines are the best fits using the "One binding site" model, with the derived dissociation constants (K_D) being indicated. Further experimental details are provided in Table S6.

.

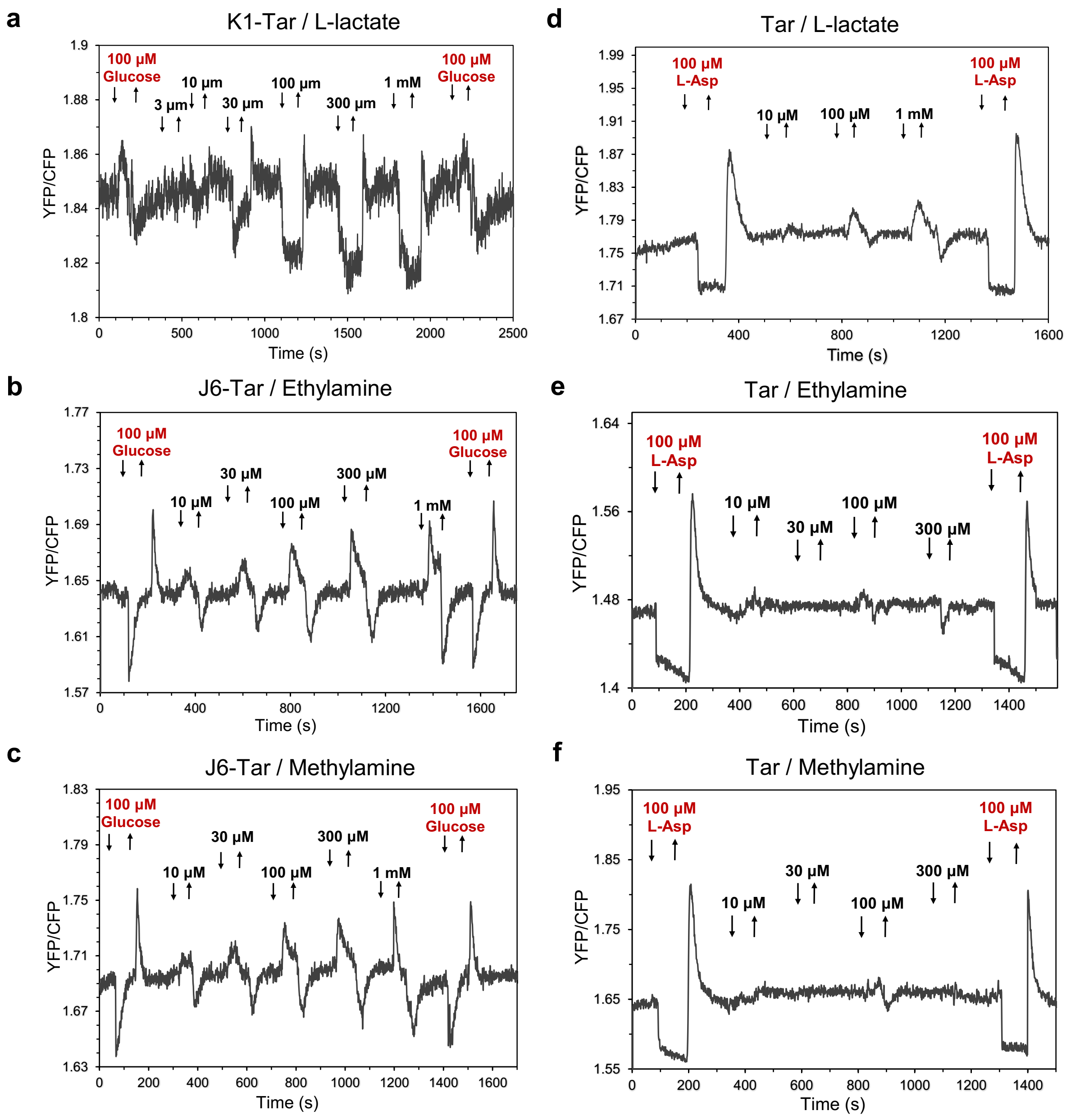

Fig. S4 Confirmation of signal transduction mediated by the K1-Tar and J6-Tar chimeras using FRET. a, FRET measurement of the K1-Tar response to L-lactate. Buffer-adapted *E. coli* cells expressing the CheZ-CFP/CheY-YFP FRET pair and K1-Tar as the sole receptor stimulated by stepwise addition (down arrow) and subsequent removal (up arrow) of the indicated concentrations of L-lactate. **b-c**, FRET measurement of the J1-Tar response to ethylamine (b) and methylamine (c). D-glucose were used as a positive control for these two hybrid chemoreceptors. **d-f**, FRET measurement of the wild-type Tar response to L-lactate (d), ethylamine (e), and methylamine (f).

Organism and protein ID	Alignment	Ligands
Key residues in K1	101 Y, 103 W, 114 L, 135 M, 153 Y, 155 F, 166 K	
V.parahaemolyticus|4EXO	DGYFFA(8)LHAIK(13)ENGVAVI(14)YFSWHKP(7)KL	Pyruvate
P.syringae|PscD|5G4Z	NDYFWI(8)MHPTN(13)PDGFAVF(15)NYRWPKP(7)KT	Glycolate, etc.
A.dehalogenans|Adeh_3718|4K08	SEYFWV(8)MHPTN(13)PNGKLLF(15)DYLWPKP(7)KI	Acetate
P.putida|McpP	DDYFWI(8)MHPAN(13)PDGFAVF(15)NYRWPKP(7)KT	Acetate, etc.
S.meliloti|McpV	SGYFWV(8)MHPIK(13)PNGKFLF(15)DYYWPKP(7)KY	Propionate, etc.
▸ *R.intestinalis*|A7	SGYFWI(8)MHPIL(13)QNGVKII(16)EFYFTKA(7)KI	Pyruvate ◂
▸ *R.faecis*|I4	AGYFWI(8)VL-LG(13)AKGYQMV(15)DYVFPKE(7)KR	L-lactate ◂
▸ *R.intestinalis*|A8	AGYFWV(8)VL-LG(13)ANGYQMV(15)DYVFPKE(7)KR	L-lactate ◂
▸ *L.pectinoschiza*|K1	AGYFWV(8)VL-LG(13)ADGYQMV(15)DYVFPKE(7)KR	L-lactate ◂
▸ *R.inulinivorans*|C1	NGYFWV(8)VL-LG(13)TNGFAYM(15)DYVFPRE(7)KR	L-lactate ◂

Fig. S5 Multiple sequence alignment of selected sCache_2 domains specific for short-chain carboxylic acids (SCCAs). The conserved residues were highlighted in pink. Newly characterized sensory domains are marked with triangles. The numbers above the alignment correspond to the amino acid positions in K1.

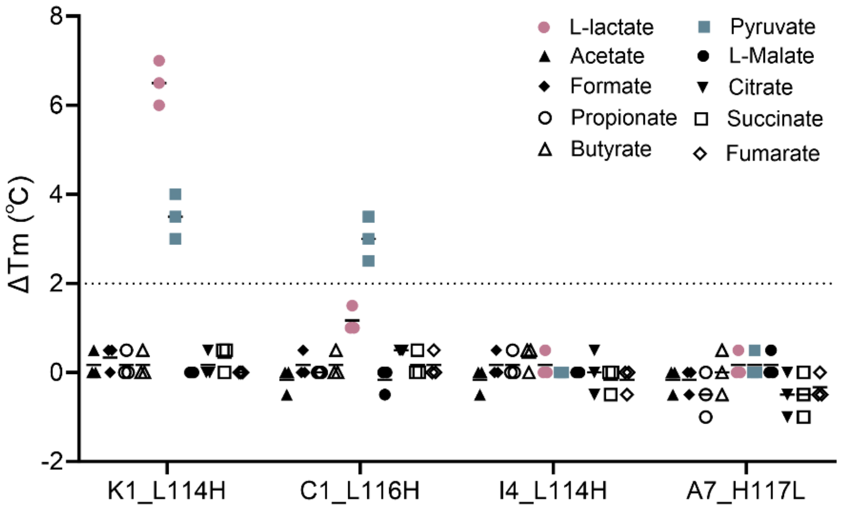

Fig. S6 Binding studies of four mutated sCache_2 SCCA domains. Theram shift measurements for sensor proteins mutants with 2 mM concentrations of indicated short-chain carboxylic acids (SCCAs). Each data point represents the individual biological measurement. Short black lines represent the mean of three independent biological replicates. The black dashed line indicates the threshold of 2 °C used as a significance cutoff for ligand identification.

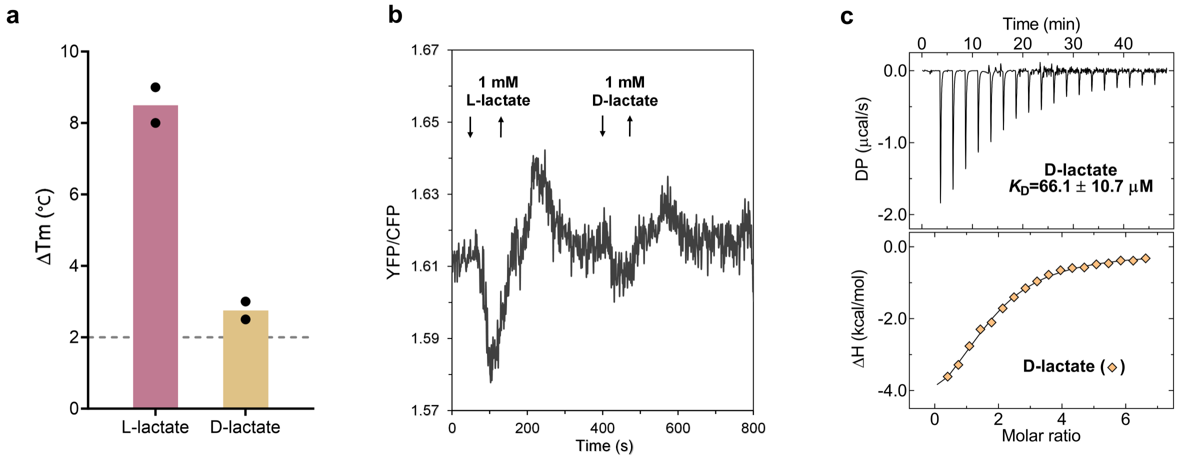

Fig. S7 Characterization of K1 sensory domain response to D-lactate. **a**, Thermal shifts observed for the K1 sensory domain upon exposure to 2 mM of either L-lactate or D-lactate. Data represent the mean of two biological replicates (indicated by dots). **b**, FRET measurements of the K1-Tar chimera response to 1 mM L-lactate or D-lactate. Buffer-adapted *E. coli* cells expressing the CheZ-CFP/CheY-YFP FRET pair and the K1-Tar chimera as the sole receptor responded to stepwise addition (down arrow) and subsequent removal (up arrow) of stimui. **c**, Measurement of the binding affinity of K1 sensory domain to D-lactate using ITC. The upper panel shows raw titration data, and the lower shows integrated corrected peak areas of the titration data fitted using the "One binding site" model. The derived dissociation constant is shown in the upper panel. Further experimental details are provided in Table S6.

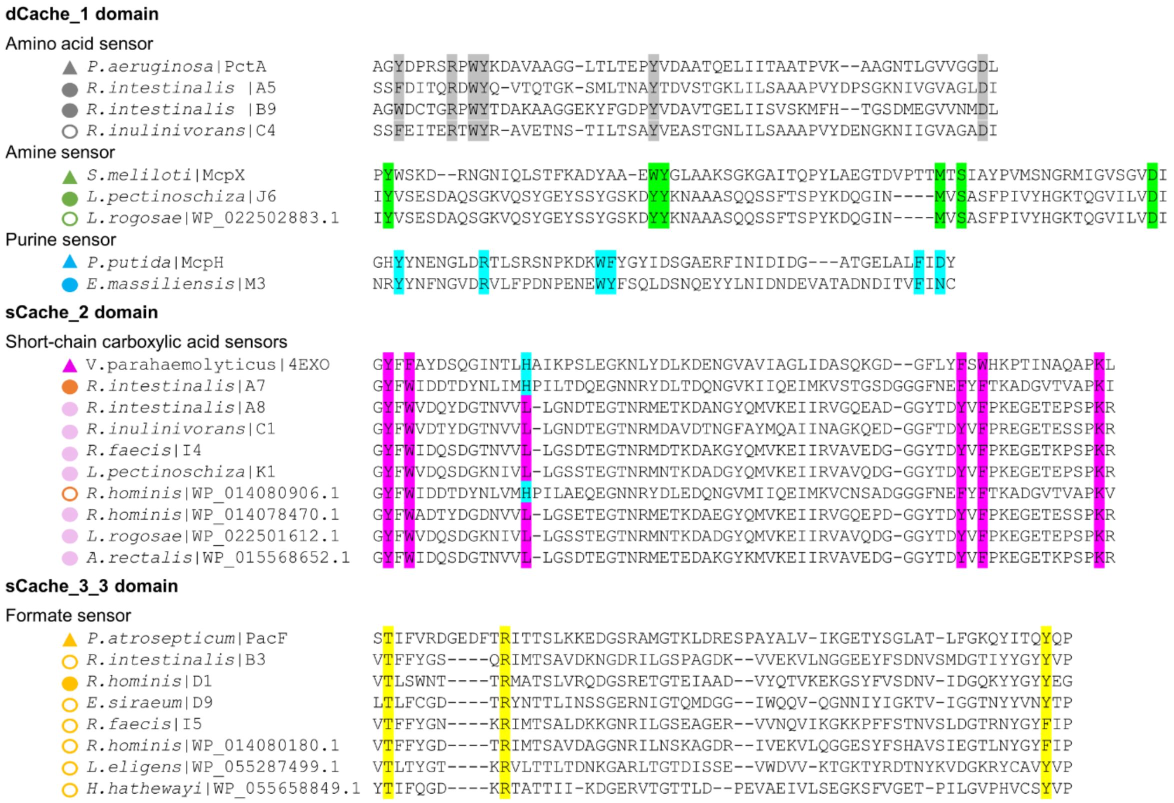

Fig. S8 Multiple sequence alignments for LBDs containing characterized ligand-binding motifs. The key ligand-binding residues (motifs) were identified through sequence alignments with previously well-characterized sensory domains, including PctA (an amino acid sensor from *Pseudomonas aeruginosa*), McpX (an amine sensor from *Sinorhizobium meliloti*), McpH (a purine sensor from *Pseudomonas putida*), 4EXO (a short-chain carboxylic acid sensor from *Vibrio parahaemolyticus*), and PacF (a formate sensor from *Pectobacterium atrosepticum*). These previously characterized LBDs are labeled with triangles. LBDs labeled with solid circles denote the newly experimentally identified sensors in this study, while those labeled with open circles denote putative sensors predicted to bind the indicated ligands. Distinct sensor clusters are color-coded based on ligand specificity: gray for amino acid, green for amines, blue for purines, orange for pyruvate, pink for lactate, and yellow for formate.

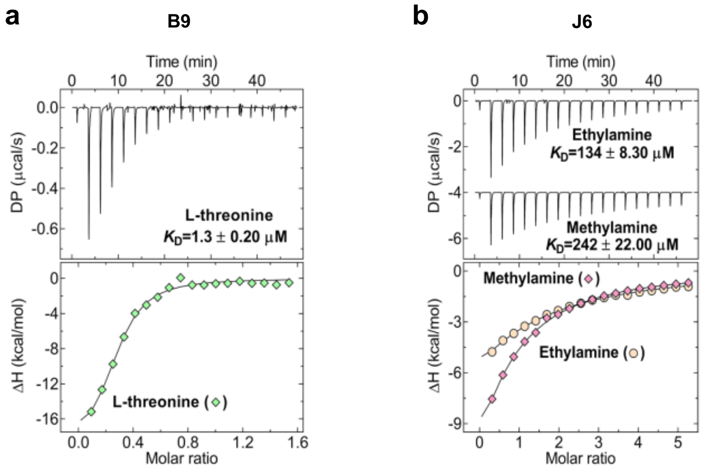

Fig. S9 ITC measurements of ligand binding to dCache domains. **a**, Binding of B9 LBD to L-threonine. **b**, Binding of J6 LBD to indicated ligands. Upper panels: Raw titration data. Lower panels: Integrated, dilution heat corrected, and concentration normalized raw data. The lines are the best fits using the "One binding site" model, with the derived dissociation constants (K_D) being indicated. Further experimental details are provided in Table S6.

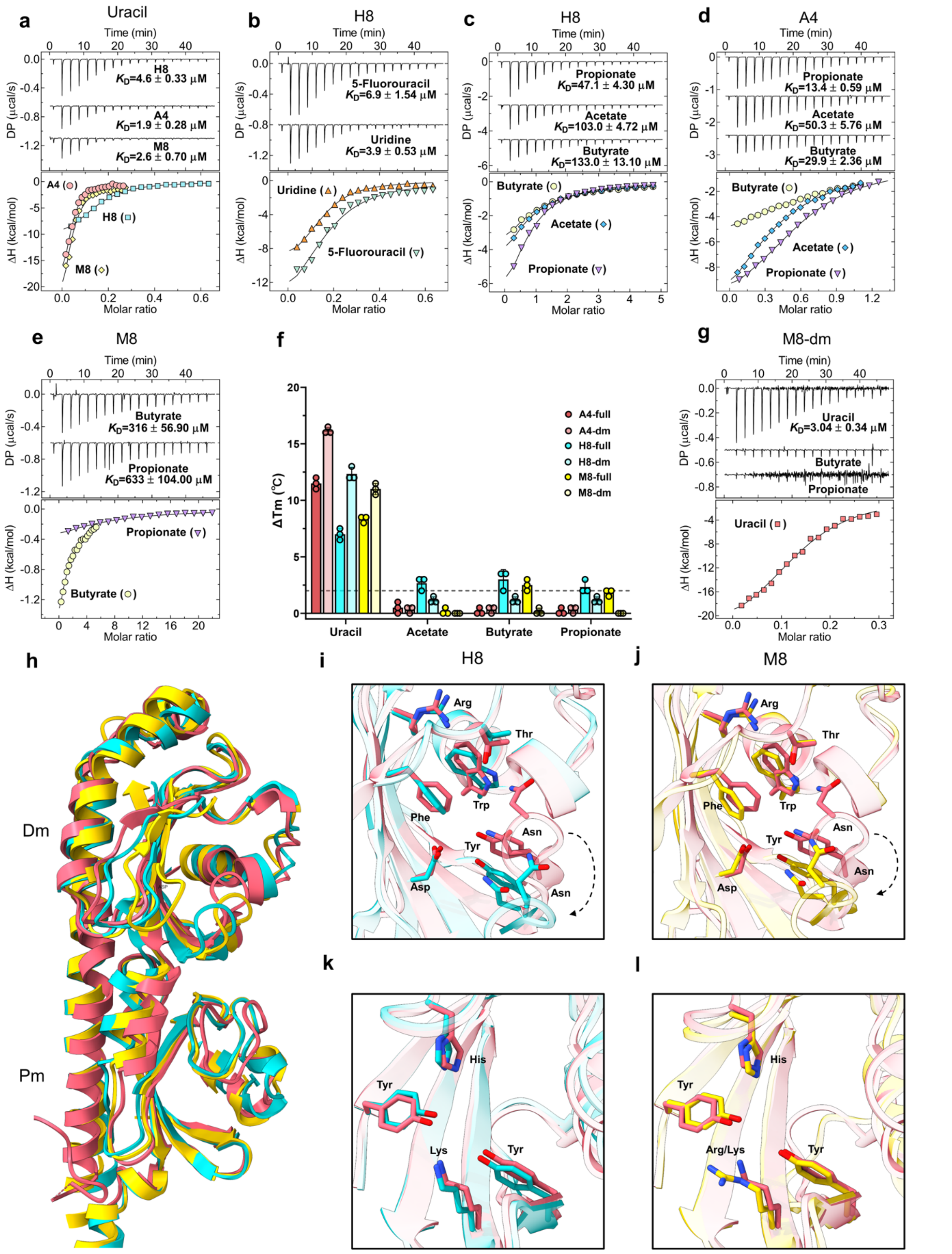

Fig. S10 Characterization of uracil and short-chain fatty acid binding to dCache_1UR domains. **a**, ITC studies of uracil binding to the H8 LBD, M8 LBD, and A4 LBD. Upper panels: Raw titration data. Lower panels: Integrated, dilution heat corrected, and concentration normalized raw data. The lines are the best fits using the "One binding site" model, with the derived dissociation constants (K_D) being indicated. **b**, ITC studies of H8 LBD with uridine and 5-fluorouracil. **c-e**, ITC studies of H8 LBD (c), A4 LBD (d), and M8 LBD (e) with the indicated short-chain fatty acids (SCFAs). **f**, Thermal shift measurements for the full-length (full) and membrane-distal module (dm) of H8, M8, and A4 in the

presence of 2 mM concentrations of the indicated compounds. Data are presented as the mean ± standard deviation from three replicates, with each point indicates an individual measurement. **g**, ITC studies of M8-dm with uracil, butyrate, and propionate. Further experimental details are provided in Table S5. **h**, The overall structural superimposition of the A4 structure with AlphaFold3 models of H8 and M8. Three uracil sensors are depicted in different colors: A4 - red, H8 - cyan, and M4 - yellow. **i**, **j**, Structural alignment of uracil binding pocket of A4 with the membrane-distal modules of H8 AlphaFold3 model (i) and M8 AlphaFold3 model (j). The amino acids in H8 and M8 corresponding to the uracil-binding key residues of A4 are labeled. **k**, **l**, Structural alignment of acetate binding pocket of A4 with the membrane-proximal modules of H8 AlphaFold3 model (k) and M8 AlphaFold3 model (l). The amino acids in H8 and M8 corresponding to the SCFA binding key residues of A4 are labeled. The dashed arrow indicates a loop displacement between the crystallographic structure of A4 and the AlphaFold3 models of H8 and M8.

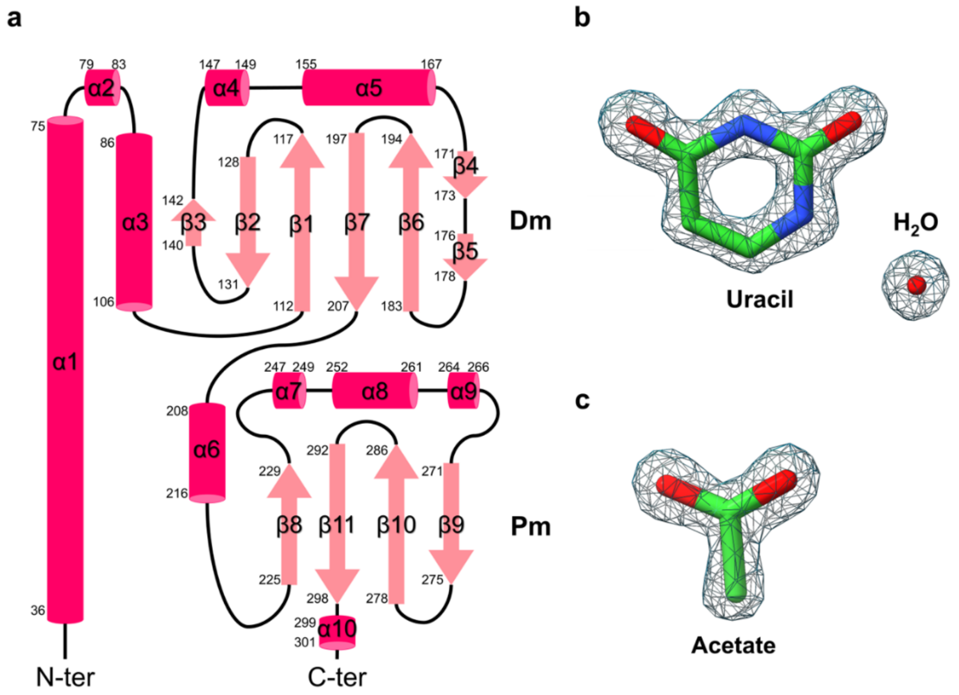

Fig. S11 Topology of the A4 LBD and electron density of ligands. a, Topology of the A4 LBD secondary elements. The α-helices are represented by cylinders and the β-sheets by arrows. Dm and Pm stand for membrane-distal module and membrane-proximal module, respectively. **b**, **c**, 2mFo-DFc electron density maps of uracil and a water molecule (b), and acetate (c). 2mFo-DFc map (gray mesh) is contoured at 1.24σ.

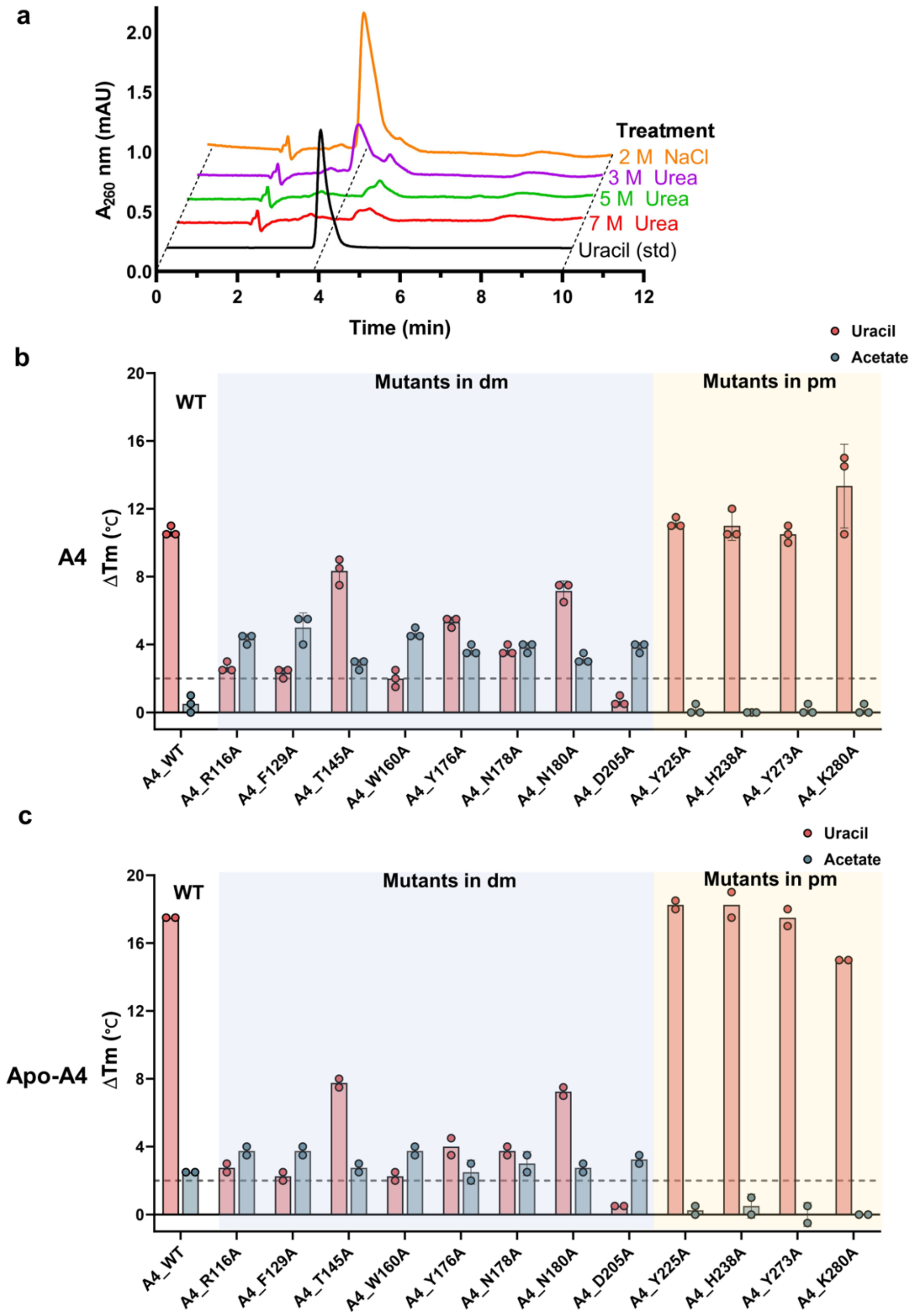

Fig. S12 Experimental evaluation of the contributions of individual key amino acids in the A4 LBD to ligand binding. a, HPLC chromatogram of the A4 LBD after different treatments to remove endogenously bound ligands. A solution containing only uracil (black trace) was used as a standard. **b**, Thermal shift measurements for the indicated A4 LBD mutants in the presence of 2 mM uracil or acetate. The data represent the mean ± standard deviations of three biological replicates. **c**, Thermal shift measurements for the apo-A4 BD mutants in the presence of of 2 mM uracil or acetate. The data represent the means of two biological replicates. Data are categorized into three groups: wild-type A4 (WT), A4 with mutations in the membrane-distal module (mutants in dm; blue), and A4 with mutations in the membrane-proximal module (mutants in pm; yellow). Each data point represents an independent biological measurement. The gray dashed line indicates the threshold of 2°C used as a significance cutoff for ligand identification.

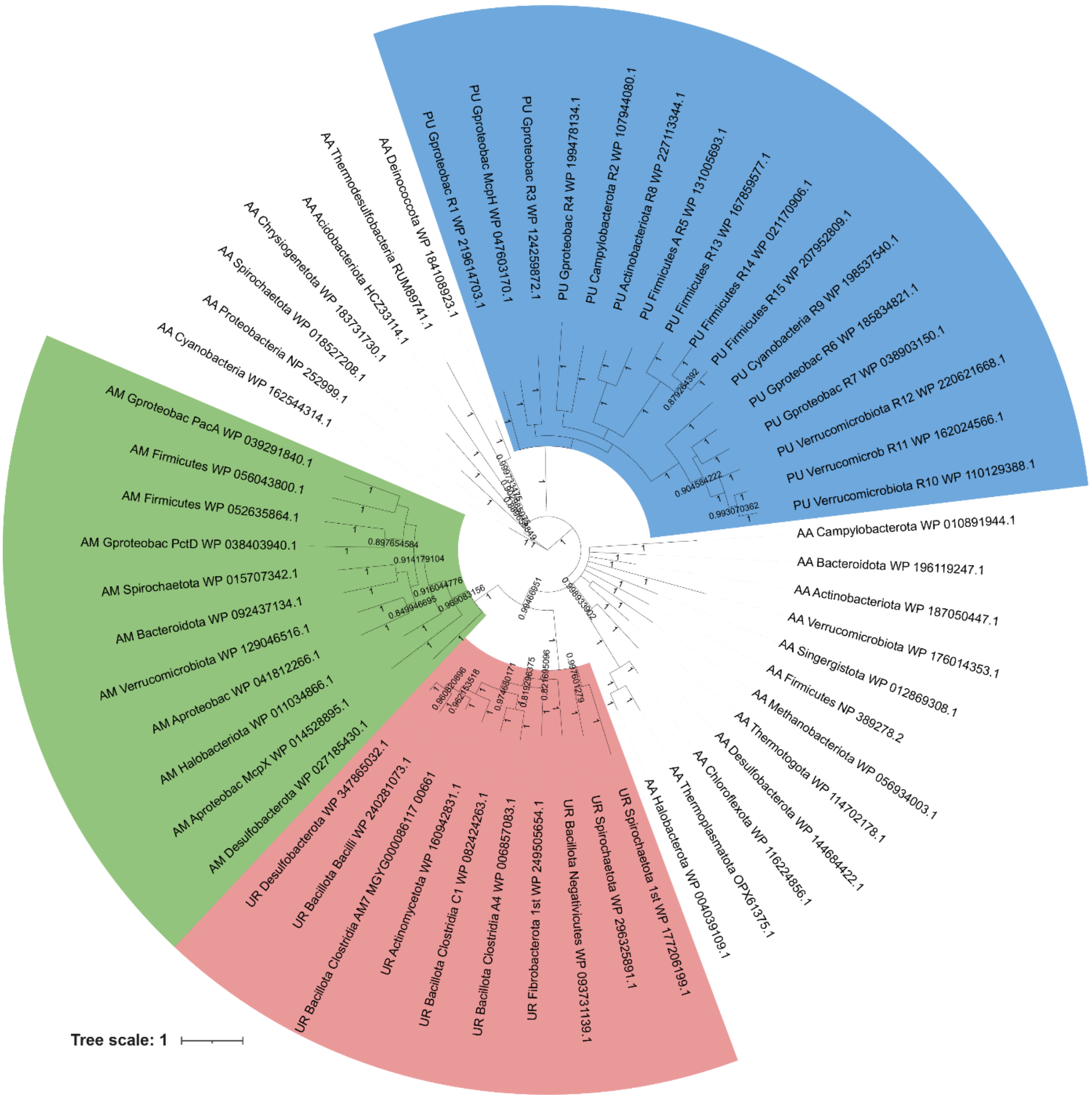

Fig. S13 Bayesian phylogenetic tree depicted in Fig. 5b with annotation of individual proteins. Colors and abbreviations are as in Fig. 5b.

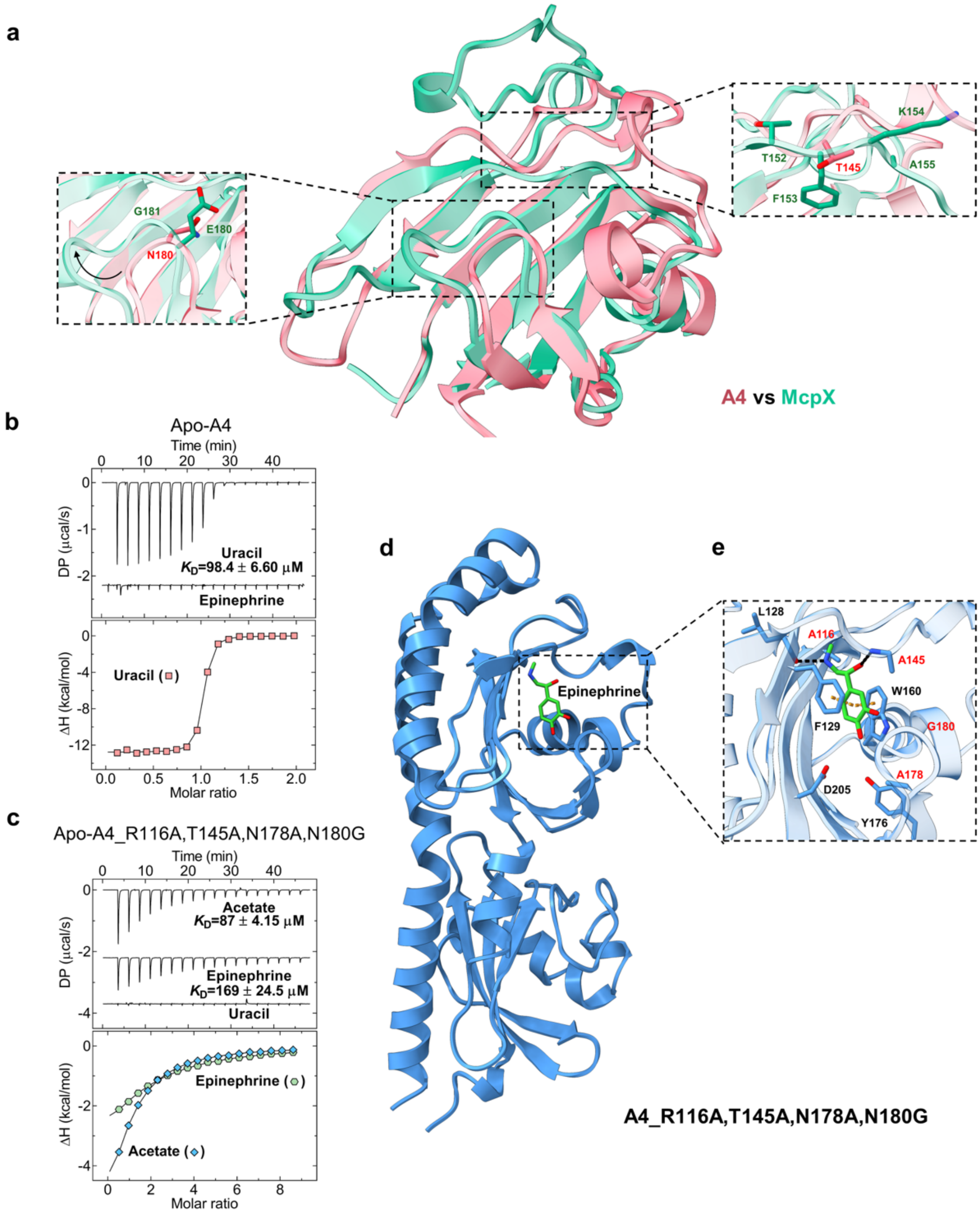

Fig. S14 Binding of epinephrine to the A4_R116A, T145A, N178A, N180G LBD mutant. a, Structural superimposition of the membrane-distal module of the A4 (red) and McpX (green) LBDs. Close-up views highlight T145 and N180 in A4, along with their corresponding residues in McpX, as determined through structural (F153 and E180) and sequence alignments (A155 and G181). **b**, **c**, ITC measurements of Apo-A4 LBD (b) and Apo-A4_R116A, T145A, N178A, N180G mutant LBD (c) binding to uracil and epinephrine. The upper panel shows raw titration data, and the lower shows integrated corrected peak areas of the titration data fitted using the "One binding site" model. The derived dissociation constant is shown in the upper panel. Further experimental details are provided in Table S5. **d**, **e**, Computational docking analysis of the Apo-A4_R116A, T145A, N178A, N180G mutant with epinephrine. The overall structure of the Apo-A4_R116A, T145A, N178A, N180G mutant LBD (generated by PyMOL) with epinephrine is illustrated in panel (d), with a magnified view of the ligand binding site provided in panel (e). Key residues predicted to interact with epinephrine are depicted in stick mode. Predicted interactions are depicted in dashed lines: orange for π-π stacking and black for hydrogen bonds. The mutated residues are highlighted in red.

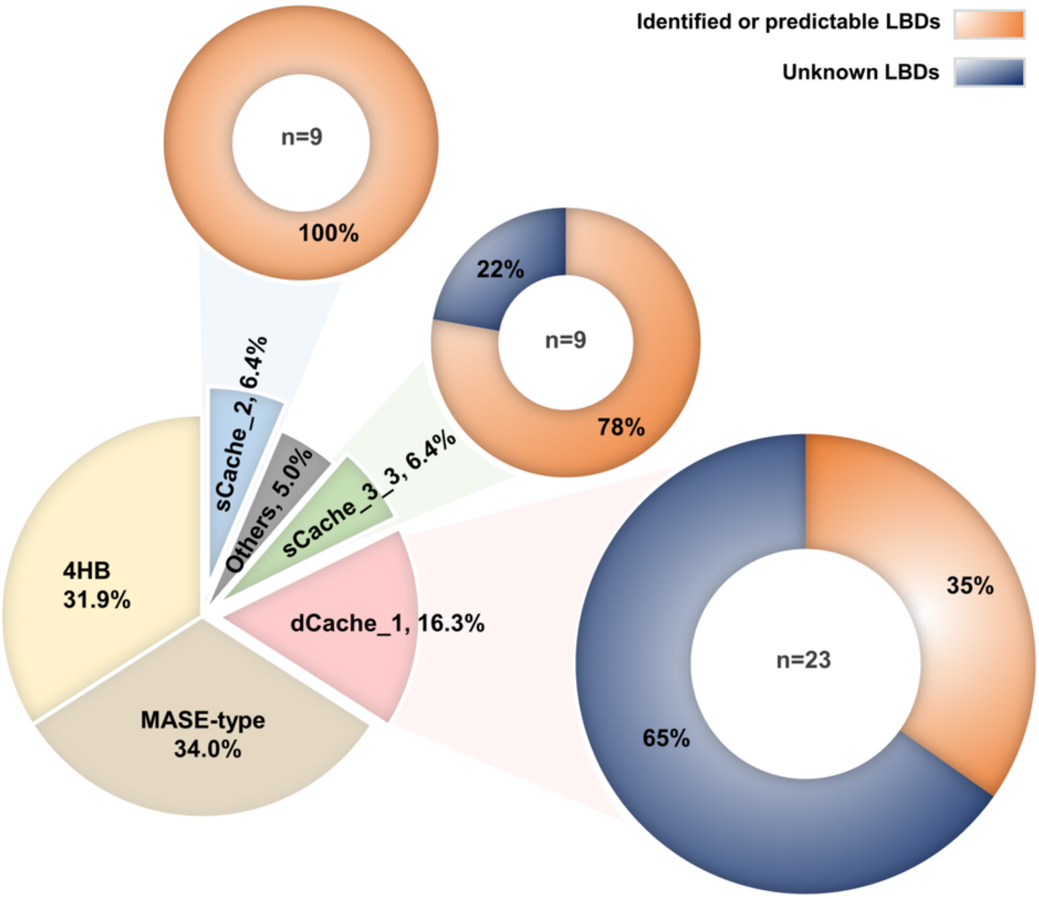

Fig. S15 Overview of MCP specificity based on experimental identification and bioinformatic predictions. The 12 gut bacterial species analyzed possess 141 transmembrane MCPs with assigned LBDs, with the full repertoire of MCPs presented in Dataset S3. Approximately one-third of these LBDs belong to the 4HB superfamily, another third to the MASE-type, and the remaining third to the Cache superfamily, which is further divided into four subgroups. Ligands of LBDs were identified experimentally or predicted using bioinformatics. All sCache_2 domains bind short-chain carboxylic acids. For sCache_3_3 domains, 78% have ligands identified through experiments or predictions based on the known formate sensors. For dCache_1 domains, 35% have ligands identified experimentally or through conserved binding motifs for amino acids, amines, or purines. Supporting details are available in Dataset S3 and Fig. S6.

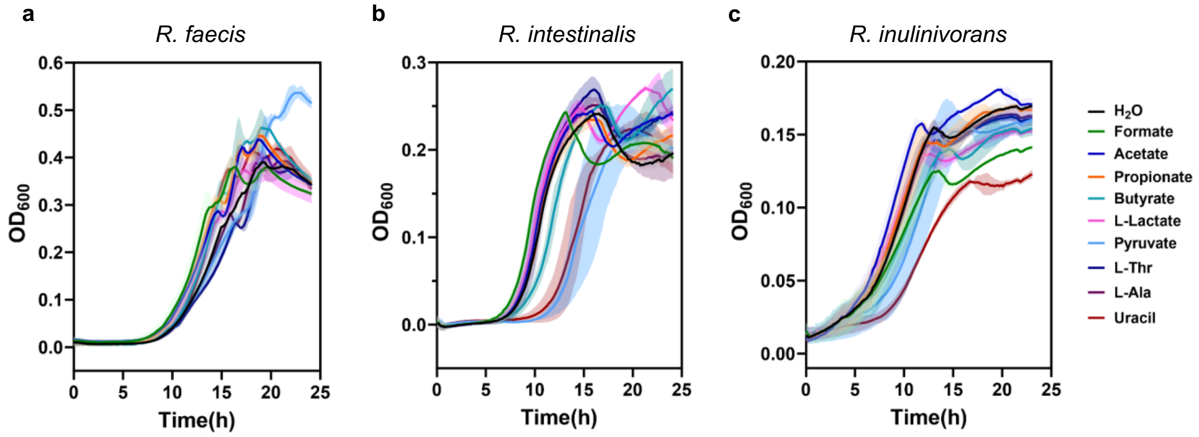

Fig. S16 Effects of chemoeffector compounds on growth of selected *Roseburia* species. a-c, Growth curves of *R. faecis* (a), *R. intestinalis* (b), and *R. inulinivorans* (c) in presence of 20 mM of indicated compounds (10 mM for uracil) added to 50% YCFA medium (see Methods). Growth was assessed by measuring optical density (OD) at 600 nm (OD_{600}). Solid lines represent the mean values from three biological replicates, with shaded areas indicating the standard deviations. Data represent mean values from three biological replicates.

SI Tables

Table S1. Source gut bacteria of the studied sensory domains.

NCBI Organism name[1]	GTDB Taxonomy	Genome Accession	No.[2]
Agathobacter rectalis	d__Bacteria; p__Bacillota_A; c__Clostridia; o__Lachnospirales; f__Lachnospiraceae; g__Agathobacter; s__Agathobacter rectalis	GCF_001404855.1	6
[Eubacterium] siraeum DSM 15702	d__Bacteria; p__Bacillota_A; c__Clostridia; o__Oscillospirales; f__Ruminococcaceae; g__Ruminiclostridium_E; s__Ruminiclostridium_E siraeum	GCF_000382085.1	4
Blautia obeum ATCC 29174	d__Bacteria; p__Bacillota_A; c__Clostridia; o__Lachnospirales; f__Lachnospiraceae; g__Blautia_A; s__Blautia_A obeum	GCF_000153905.1	2
Blautia wexlerae DSM 19850	d__Bacteria; p__Bacillota_A; c__Clostridia; o__Lachnospirales; f__Lachnospiraceae; g__Blautia_A; s__Blautia_A wexlerae	GCF_000484655.1	17
Catenibacterium mitsuokai DSM 15897	d__Bacteria; p__Bacillota_I; c__Bacilli_A; o__Erysipelotrichales; f__Coprobacillaceae; g__Catenibacterium; s__Catenibacterium mitsuokai	GCF_000173795.1	3
Collinsella aerofaciens	d__Bacteria; p__Actinomycetota; c__Coriobacteriia; o__Coriobacteriales; f__Coriobacteriaceae; g__Collinsella; s__Collinsella aerofaciens_A	GCF_002736145.1	1
Eisenbergiella massiliensis	d__Bacteria; p__Bacillota_A; c__Clostridia; o__Lachnospirales; f__Lachnospiraceae; g__Eisenbergiella; s__Eisenbergiella porci	GCF_900243045.1	6
Lachnospira eligens	d__Bacteria; p__Bacillota_A; c__Clostridia; o__Lachnospirales; f__Lachnospiraceae; g__Lachnospira; s__Lachnospira eligens_A	GCF_001405395.1	6
Fusicatenibacter saccharivorans	d__Bacteria; p__Bacillota_A; c__Clostridia; o__Lachnospirales; f__Lachnospiraceae; g__Fusicatenibacter; s__Fusicatenibacter saccharivorans	GCF_001406335.1	1
Hungatella hathewayi	d__Bacteria; p__Bacillota_A; c__Clostridia; o__Lachnospirales; f__Lachnospiraceae; g__Hungatella; s__ Hungatella effluvii	GCF_001405675.1	1
Lachnospira pectinoschiza	d__Bacteria; p__Bacillota_A; c__Clostridia; o__Lachnospirales; f__Lachnospiraceae; g__Lachnospira; s__Lachnospira pectinoschiza_A	GCF_001405835.1	15
Lactobacillus rogosae	d__Bacteria; p__Bacillota_A; c__Clostridia; o__Lachnospirales; f__Lachnospiraceae; g__Lachnospira; s__Lachnospira pectinoschiza_A	GCF_900112995.1	5
Phascolarctobacterium faecium DSM 14760	d__Bacteria; p__Bacillota_C; c__Negativicutes; o__Acidaminococcales; f__Acidaminococcaceae; g__Phascolarctobacterium; s__Phascolarctobacterium faecium	GCF_003269275.1	1
Roseburia faecis	d__Bacteria; p__Bacillota_A; c__Clostridia; o__Lachnospirales; f__Lachnospiraceae; g__Agathobacter; s__Agathobacter faecis	GCF_001405615.1	19
Roseburia hominis A2-183	d__Bacteria; p__Bacillota_A; c__Clostridia; o__Lachnospirales; f__Lachnospiraceae; g__Roseburia; s__Roseburia hominis	GCF_000225345.1	6
Roseburia intestinalis L1-82	d d__Bacteria; p__Bacillota_A; c__Clostridia; o__Lachnospirales; f__Lachnospiraceae; g__Roseburia; s__Roseburia intestinalis	GCF_000156535.1	11
Roseburia inulinivorans DSM 16841	d__Bacteria; p__Bacillota_A; c__Clostridia; o__Lachnospirales; f__Lachnospiraceae; g__Roseburia; s__Roseburia inulinivorans	GCF_000174195.1	8
Ruminococcus bicirculans	d__Bacteria; p__Bacillota_A; c__Clostridia; o__Oscillospirales; f__Ruminococcaceae; g__Hominimerdicola; s__Hominimerdicola aceti	GCF_000723465.1	1
Ruminococcus bromii	d__Bacteria; p__Bacillota_A; c__Clostridia; o__Oscillospirales; f__Acutalibacteraceae; g__Ruminococcus_E; s__Ruminococcus_E bromii_A	GCF_900101355.1	1
Subdoligranulum sp. 4_3_54A2FAA	d__Bacteria; p__Bacillota_A; c__Clostridia; o__Oscillospirales; f__Ruminococcaceae; g__Ruthenibacterium; s__Ruthenibacterium lactatiformans	GCF_000238635.1	2

[1]The NCBI organism name was used throughout this study to refer to the related species.
[2]The number of sensor proteins studied from the specified organism is indicated.

Table S2. Layouts of the human gut metabolite plates (HGMT plates). All compounds were dissolved in H_2O at the final concentration of 20 mM and pH 7.0, unless otherwise stated. Different chemical categories are highlighted with color.

HGMT Plate_1

	A	B	C	D	E	F	G	H
1	H_2O	L-Lysine	D-Aspartic acid	Ethanolamine	Acetylcholine	Pyruvate	4-Methylaminobutyric acid	D-Quinic acid
2	L-Alanine	L-Methionine	D-Serine	L-Carnitine	Choline	L-Malate	Glutathione reduced	Ferulic acid
3	L-Arginine	L-Phenylalanine	Ala-Gln	Betaine	Isobutyric acid	Fumarate	3-Hydroxypropionic acid	Trans-Aconitic acid
4	L-Asparagine	L-Proline	Gly-Glu	Spermidine[a]	Formate	Tartrate	β-Hydroxybutyric acid	α-Ketoglutaric acid
5	L-Aspartic acid	L-Serine	N-Acetyl-L-Glutamate (GluNAC)	Spermine[a]	Acetate	Oxalacetic acid	5-Aminovalerate	
6	L-Cysteine	L-Threonine	N-Acetyl-L-aspartic acid	Agmatine	Propionate	Glutaric acid	Methylmalonic acid	
7	L-Glutamine	L-Tryptophan	Gly-Gly-Ala	Putrescine	Isovaleric acid	L-Lactate	Tricarballylate	
8	L-Glutamic acid	L-Tyrosine (KOH)	Creatine Monohydrate	Phenethylamine	Butyrate	Succinate	α-Aminobutyric acid	
9	L-Glycine	L-Valine	Dimethylamine hydrochloride	Histamine	Citrate	Phenylacetate	Glyoxylic acid monohydrate	
10	L-Histidine	L-Ornithine	Methylamine hydrochloride	Trimethylamine	Gluconic acid	Salicylate	γ-aminobutyric acid	
11	L-Isoleucine	L-Citrulline	Tyramine hydrochloride	Ethylenediamine	Malonate	Caffeic acid	Maleate	
12	L-Leucine	D-Alanine	Trimethylamine N-oxide (TMAO)	Ethylamine	Oxalic acid	Itaconate	Shikimic acid	

HGMT Plate_2

	A	B	C	D	E	F
1	H_2O	L-Fucose	D-Trehalose	Sodium cholate[b]	Hypoxanthine (10 mM)	Inosine
2	D-Xylose	α-D-Raffinose·H_2O	Inulin	Taurine	Adenosine	Uridine
3	D-Mannitol	D-Galactose	L-Arabinose	Urea	Purine	Cytosine (10 mM)
4	D-Fructose	Glucosamine	Ascorbate	Acetamide	Uric acid (50 mM KOH)	Theophylline
5	D-Maltose	Methyl-α-D-Glucopyranoside	Biotin	$NaNO_2$	Xanthine (50 mM KOH)	Thymidine
6	Lactose	D-Glucose-6-phosphate	Vitamin B1 (Thiamin)	$NaNO_3$	Guanine (50 mM KOH)	
7	D-Mannose	D-Ribose	Myo-inositol	Indole (10 mM)	Guanosine (50 mM KOH)	
8	D-Glucose	N-Acetyl-D-Glucosamine (GlcNAC)[a]	Nicotinic acid	Allantoin	Adenine (10 mM)	
9	D-Sucrose	N-Acetyl-D-Galactose (GalNAC)	Nicotinamide	KOH (50 mM)	Uracil (10 mM)	
10	Xylan	D-Fructose-6-phosphate[a]	Pyridoxine hydrochloride	(±)-Epinephrine hydrochloride	Thymine (10 mM)	
11	D-Sorbitol	D-Ribose-5-phosphate[a]	Hyocholic acid (10 mM)	Dopamine hydrochloride	Cytidine	
12	L-Rhamnose	Isomaltulose	Sodium taurocholate hydrate[b]	3,4-Dihydroxymandelic acid	Caffeine	

Amino acids and derivatives	Biogenic amines	Short-chain fatty acids	Other carboxylic acids	Sugars and derivatives
Vitamins	Bile acids	Hormones	Nucleobases and derivatives	Other compounds

[a] Compounds that caused thermal shifts in multiple ligand binding domains, possibly because these ligands induce nonspecific conformational changes in LBDs.

[b] Compounds that caused protein unfolding in thermal shift assays.

Table S3. Putative ligands identified in the initial high-throughput ligand screening via TSA.

	Ligand[a]	Sensory domain	Domain family	Source organism
**Amino acids**	L-arginine	B9	dCache_1	*Roseburia inulinivorans*
	L-valine			
	L-threonine			
	L-glycine	A5		*Roseburia intestinalis* L1-82
	L-alanine			
	D-serine			
**Biogenic amines**	Methylamine	J6		*Lachnospira pectinoschiza*
	Ethylamine			
**Nucleobases and their derivatives**	Inosine	M3		*Eisenbergiella massiliensis*
	Hypoxanthine			
	Theophylline			
	Uracil	H8, A4, M8		*Roseburia faecis*, *Roseburia intestinalis* L1-82, *Hungatella hathewayi*
	Uridine			
**C3/C4-dicarboxylic acids**	Succinate	D8		*[Eubacterium] siraeum*
	Itaconate			
	Maleate			
	Methylmalonate			
**Short-chain fatty acids**	Formate	D1	sCache_3_3	*Roseburia hominis* A2-183
	Acetate	H8	dCache_1	*Roseburia faecis*, *Roseburia intestinalis* L1-82, *Hungatella hathewayi*
	Propionate	H8, M8		
	Butyrate	H8, M8		
**Other short-chain carboxylic acids**	Pyruvate	A7	sCache_2	*Roseburia intestinalis* L1-82
	L-lactate	A8, I4, C1, K1		*Roseburia intestinalis* L1-82, *Roseburia faecis*, *Roseburia inulinivorans*, *Lachnospira pectinoschiza*
**Sugar**	D-fructose	K1		*Lachnospira pectinoschiza*
**Indole**	Indole	B6	SMP_2	*Catenibacterium mitsuokai*

For each ligand, the sensory domain that recognizes it, the domain family that the corresponding sensory domain belongs to, and the organism from which the corresponding sensory domain comes are shown.

[a] Ligands that can induce thermal shift over 2 °C.

Table S4. Reported concentrations of selected metabolites in the mammalian intestine.

Metabolite	Ligand	Sample source	Concentration	Ref.
Amino acids	L-arginine	Human, intestinal lumen	Free amino acid concentration: 50-300 μM, 0.6-6 mM after a protein-rich meal	(26)
	L-valine			
	L-threonine			
	L-glycine			
	L-alanine			
	D-serine			
Biogenic amines	Methylamine	Human, colon	Proximal colon: 0-6.0 mM; distal colon: 2.2-3.2 mM	(27)
	Ethylamine	-	-	-
Nucleobases and their derivatives	Inosine	Mice, GI tract	Duodenum: 66.13±14.23 μM; jejunum: 29.26±9.38 μM; cecum 0.5±0.05 μM	(28)
	Hypoxanthine	Mice, small intestine	0-0.3 mM	(29)
	Theophylline	Rabbit, GI tract	Up to10 mM (depending on diet)	(30)
	Uracil	Mice, small intestinal	0-0.5 mM	(29)
C3/C4-dicarboxylic acids	Succinate	Human, feces	6.3±1.7 mM (in healthy control)	(31)
	Itaconate	-	-	
	Maleate	-	-	
	Methylmalonate	-	-	
Short-chain fatty acids	Formate	Human, colon	0-5mM	(32)
	Acetate	Human, feces	17.9-164.1 mM; average: 62.4 mM	(33)
	Propionate	Human, feces	4.3-49.8 mM; average: 21.0 mM	(33)
	Butyrate	Human, feces	1.6-70.1 mM; average: 18.8 mM	(33)
Other short-chain carboxylic acids	Pyruvate	-	-	-
	L-lactate	Human, feces	0-25 mM	(34)
Sugar	D-fructose	Human, small intestine	6-15 mM (under fasting conditions)	(35)
Indole	Indole	Human, GI tract	0.2-6.5 mM	(36)

Table S5. Strains, plasmids, and oligonucleotides used in this study.

Strains and plasmids	Genotype or relevant characteristics[a]	Reference
Strains		
T7 Express (Enhanced *E. coli* BL21 derivative)	*fhuA2 lacZ::T7 gene1 [lon] ompT gal sulA11 R(mcr-73::miniTn10*--Tet^S*)2 [dcm] R(zgb-210::Tn10*--Tet^S*) endA1 Δ(mcrC-mrr)114::IS10*	New England Biolabs, (37)
E. coli DH5α	F⁻ φ80*lac*ZΔM15 Δ(*lac*ZYA-*arg*F) U169 *rec*A1 *end*A1 *hsd*R17(r_K^-, m_K^+) *pho*A *sup*E44 λ⁻*thi*-1 *gyr*A96 *rel*A1	(38)
E. coli UU1250	Derivative of RP437; Δ*aer*Δ*tsr*Δ(*tar-tap*) Δ*trg*	(39)
E. coli VS181	Derivative of RP437; Δ(*cheYcheZ*)Δ*aer*Δ*tsr*Δ(*tar-tap*) Δ*trg*	(40)
Plasmids		
pET28a (+)	Km^R; Protein expression vector	Novagen
pKG116	Cm^R; Expression vector, salicylate inducible; for generation of hybrid chemoreceptor	(41)
pSB13	Cm^R; Tar expression plasmid, pKG116 derivative, T768 was mutated to C768 to remove the NdeI restriction site	(42)
pVS88	Ap^R; CheY-EYFP / CheZ-ECFP expression plasmid	(40)
pET28_LBDs library	Km^R; pET28a (+) derivative containing a DNA fragment encoding individual full-length LBD from LBD library	This study
pET28_K-Y101A	Km^R; pET28a (+) derivative containing a DNA fragment encoding the $K1_{LBD}$-Y101A mutant	This study
pET28_K1-W103A	Km^R; pET28a (+) derivative containing a DNA fragment encoding the $K1_{LBD}$-W103A mutant	This study
pET28_K1-L114A	Km^R; pET28a (+) derivative containing a DNA fragment encoding the $K1_{LBD}$-L114A mutant	This study
pET28_K1-M135A	Km^R; pET28a (+) derivative containing a DNA fragment encoding the $K1_{LBD}$-M135A mutant	This study
pET28_K1-Y153A	Km^R; pET28a (+) derivative containing a DNA fragment encoding the $K1_{LBD}$-Y153A mutant	This study
pET28_K1-F155A	Km^R; pET28a (+) derivative containing a DNA fragment encoding the $K1_{LBD}$-F155A mutant	This study
pET28_K1-K166A	Km^R; pET28a (+) derivative containing a DNA fragment encoding the $K1_{LBD}$-K166A mutant	This study

pET28_ C1-L116H	Km^R; pET28a (+) derivative containing a DNA fragment encoding the $C1_{LBD}$-L116H mutant	This study
pET28_A7-H117L	Km^R; pET28a (+) derivative containing a DNA fragment encoding the $A7_{LBD}$-H117L mutant	This study
pET28_ K1-L114H	Km^R; pET28a (+) derivative containing a DNA fragment encoding the $K1_{LBD}$-L114H mutant	This study
pET28_I4-L114H	Km^R; pET28a (+) derivative containing a DNA fragment encoding the $I4_{LBD}$-L114H mutant	This study
pET28_D8-dm	Km^R; pET28a (+) derivative containing a DNA fragment encoding the distal module of $D8_{LBD}$	This study
pET28_D8-pm	Km^R; pET28a (+) derivative containing a DNA fragment encoding the proximal module of $D8_{LBD}$	This study
pET28_C1-dm	Km^R; pET28a (+) derivative containing a DNA fragment encoding the distal module of $C1_{LBD}$	This study
pET28_A4-dm	Km^R; pET28a (+) derivative containing a DNA fragment encoding the distal module of $A4_{LBD}$	This study
pET28_M8-dm	Km^R; pET28a (+) derivative containing a DNA fragment encoding the distal module of $M8_{LBD}$	This study
pET28_A4-R116A	Km^R; pET28a (+) derivative containing a DNA fragment encoding the $A4_{LBD}$-R116A mutant	This study
pET28_A4-F129A	Km^R; pET28a (+) derivative containing a DNA fragment encoding the $A4_{LBD}$-F129A mutant	This study
pET28_A4-T145A	Km^R; pET28a (+) derivative containing a DNA fragment encoding the $A4_{LBD}$-T145A mutant	This study
pET28_A4-W160A	Km^R; pET28a (+) derivative containing a DNA fragment encoding the $A4_{LBD}$-W160A mutant	This study
pET28_A4-Y176A	Km^R; pET28a (+) derivative containing a DNA fragment encoding the $A4_{LBD}$-Y176A mutant	This study
pET28_A4-N178A	Km^R; pET28a (+) derivative containing a DNA fragment encoding the $A4_{LBD}$-N178A mutant	This study
pET28_A4-N180A	Km^R; pET28a (+) derivative containing a DNA fragment encoding the $A4_{LBD}$-N180A mutant	This study
pET28_A4-D205A	Km^R; pET28a (+) derivative containing a DNA fragment encoding the $A4_{LBD}$-D205A mutant	This study
pET28_A4-Y225A	Km^R; pET28a (+) derivative containing a DNA fragment encoding the $A4_{LBD}$-Y225A mutant	This study

pET28_A4-H238A	Km^R; pET28a (+) derivative containing a DNA fragment encoding the $A4_{LBD}$-H238A mutant	This study
pET28_A4-Y273A	Km^R; pET28a (+) derivative containing a DNA fragment encoding the $A4_{LBD}$-Y273A mutant	This study
pET28_A4-K280A	Km^R; pET28a (+) derivative containing a DNA fragment encoding the $A4_{LBD}$-K280A mutant	This study
pET28_A4-R116A, T145A, N178A, N180G	Km^R; pET28a (+) derivative containing a DNA fragment encoding the $A4_{LBD}$-R116A, T145A, N178A, N180G mutant	This study
J6-Tar	Cm^R; pKG116 derivative containing a DNA fragment encoding J6 [1-345]-SLLPY-Tar [203-553];	This study
K1-Tar	Cm^R; pKG116 derivative containing a DNA fragment encoding K1 [1-218]-LSVRL-Tar [203-553];	This study
K1-Tar-L114H	Cm^R; pKG116 derivative containing a DNA fragment encoding K1 [1-218]-LSVRL-Tar [203-553]; L114 was mutated to H114;	This study

[a]Ap, ampicillin; Km, kanamycin; Tc, tetracycline; Cm, chloramphenicol; Sm, streptomycin

Oligonucleotides

Oligonucleotide	Sequence (5'-3')	Purpose
K1_ Y101A_F	TGAGGCCGGCgcgTTCTGGGTCG	Construction of pET28_ $K1_{LBD}$_ Y101A
K1_ Y101A _R	CCGTAACGCATTTGG	
K1_ W103A _F	CGGCTATTTCgcgGTCGATCAATCCG	Construction of pET28_ $K1_{LBD}$_ W103A
K1_ W103A _R	GCCTCACCGTAACGC	
K1_ L114A _F	AAATATAGTGgcgCTCGGCTCGAG	Construction of pET28_ $K1_{LBD}$_ L114A
K1_ L114A _R	TTACCATCGGATTGATC	
K1_ M135A _F	TGGATATCAGgcgGTGAAAGAAATTATTCG	Construction of pET28_ $K1_{LBD}$_ M135A
K1_ M135A _R	TCGGCGTCTTTGGTG	
K1_ Y153A _F	CTATACAGATgcgGTTTTTCCGAAGGAAGGTGAAACCG	Construction of pET28_ $K1_{LBD}$_ Y153A
K1_ Y153A _R	CCCCCGCCATCCTGT	
K1_ F155A _F	AGATTACGTTgcgCCGAAGGAAGGTG	Construction of pET28_ $K1_{LBD}$_ F155A
K1_ F155A _R	GTATAGCCCCCGCCA	
K1_ K166A _F	ACCATCACCTgcgCGCAGTTACTC	Construction of pET28_ $K1_{LBD}$_ K166A
K1_ K166A _R	TCGGTTTCACCTTCC	

K1_ L114H _F	AAATATAGTGcatCTCGGCTCGAG	Construction of pET28_ K1$_{LBD}$_ L114H
K1_ L114H _R	TTACCATCGGATTGATC	
C1_ L116H _F	GAACGTGGTCcatTTGGGTAATGATAC	Construction of pET28_ C1$_{LBD}$_ L116H
C1_ L116H _R	GTCCCATCATAGGTGTC	
I4_ L114H _F	GAACGTCGTTcatCTGGGTTCGG	Construction of pET28_ I4$_{LBD}$_ L114H
I4_ L114H _R	GTGCCATCACTCTGATC	
A7_ H117L_F	CCTCATCATGctgCCGATTCTGAC	Construction of pET28_ A7$_{LBD}$_ H117L
A7_ H117L_R	TATAATCAGTATCATCAATCCAAAAG	
D8-dm_F	GTGCCGCGCGGCAGCCATATGAGTACTACGAAAGCACTGACC	Construction of pET28_ D8$_{LBD}$-dm
D8-dm_R	ACGGAGCTCGAATTCGGATCCCTAACGCACGATATCGTTCAGAA	
D8-pm_F	GTGCCGCGCGGCAGCCATATGAAAAACGGTGTTTTAAATAGTGAAG	Construction of pET28_ D8$_{LBD}$-pm
D8-pm_R	ACGGAGCTCGAATTCGGATCCCTATTTCAGGCAATGGATGGTATAAT	
H8-dm_F	GTGCCGCGCGGCAGCCATATGTCCGTACGTACCGCG	Construction of pET28_ H8$_{LBD}$-dm
H8-dm_R	ACGGAGCTCGAATTCGGATCCCTAGGTTTCATCCACCAGATCG	
A4-dm_F	GTGCCGCGCGGCAGCCATATGAGTACCTCGTATGAAGATAGCC	Construction of pET28_ A4$_{LBD}$-dm
A4-dm_R	ACGGAGCTCGAATTCGGATCCCTATGTATCGGTGAACTCGCTA	
M8-dm_F	GTGCCGCGCGGCAGCCATATGAAGACCGTCGTTGATGAAG	Construction of pET28_ M8$_{LBD}$-dm
M8-dm_R	ACGGAGCTCGAATTCGGATCCCTATTCCCGAATAACATCATAGTTG	
A4_R116A_F	CGCCTACATTgcgTATAATCCAGAATTTACG	Construction of pET28_ A4$_{LBD}$_R116A
A4_R116A_R	GTCAGGGCACCTTTC	
A4_F129A_F	AAGCGGCCTGgcgCTGACCCGTG	Construction of pET28_ A4$_{LBD}$_F129A
A4_F129A_R	GTGGGTTCCGTAAATTCTGG	
A4_T145A_F	CGTTACTCCAgcgGATTTTAGCATG	Construction of pET28_ A4$_{LBD}$_T145A
A4_T145A_R	CTCTCAAATTCACTATCCG	

A4_W160A_F	ACATGTCGGGgcgTATTACATTCCTG	Construction of pET28_ A4$_{LBD}$_W160A
A4_W160A_R	TCTACGTCGCTCGGATC	
A4_Y176A_F	GATGGAGCCTgcgCTGAACTCCAATATTG	Construction of pET28_ A4$_{LBD}$_Y176A
A4_Y176A_R	CAGGTTTCTTTACCATTCTG	
A4_N178A_F	GCCTTATCTGgcgTCCAATATTGGAGTG	Construction of pET28_ A4$_{LBD}$_N178A
A4_N178A_R	TCCATCCAGGTTTCTTTAC	
A4_N180G_F	TCTGAACTCCggcATTGGAGTGTAC	Construction of pET28_ A4$_{LBD}$_N180G
A4_N180G_R	TAAGGCTCCATCCAG	
A4_N180A_F	TCTGAACTCCgcgATTGGAGTGTAC	Construction of pET28_ A4$_{LBD}$_N180A
A4_N180A_R	TAAGGCTCCATCCAG	
A4_D205A_F	CATTGGAATGgcgATTGATTTTAGCG	Construction of pET28_ A4$_{LBD}$_D205A
A4_D205A_R	ATGCCGATAGATTCAC	
A4_Y225A_F	CGACTCTGGCgcgGGATTTCTTGTG	Construction of pET28_ A4$_{LBD}$_Y225A
A4_Y225A_R	AAAATGCTAAGACTATCAATTG	
A4_H238A_F	GGTGATGTACgcgAAAGATCTGGAAATCG	Construction of pET28_ A4$_{LBD}$_H238A
A4_H238A_R	TTTCCGGATTCATTCAC	
A4_Y273A_F	CGCGGTGAGCgcgACCTACCAGG	Construction of pET28_ A4$_{LBD}$_Y273A
A4_Y273A_R	GTTTCCTCAGTCTGTTC	
A4_K280A_F	GGGAAAGGATgcgGTGATGTATTATAAGAC	Construction of pET28_ A4$_{LBD}$_K280A
A4_K280A_R	TGGTAGGTGTAGCTC	
pKG116-seq_F	AAGCCATAAGGAGTACCATATG	Sequencing for hybrid Chemoreceptor
pKG116-seq_R	TTACTTATTTATCCGCGGATC	
pET28a-seq_F	GTGCCGCGCGGCAGCCATATG	Sequencing for LBD expression plasmids
pET28a-seq_R	ACGGAGCTCGAATTCGGATCCCTA	
J6-Tar_F	AGCCATAAGGAGTACCATATGATGAGCAAAGAAC ACACG	Construction of J6-Tar chimera
J6-Tar _R	CCACCAGCAGAATCAANNNNNNNNNNNNNNNAA CAATCATAAATACCACTAGAATA	
K1-Tar_F	AGCCATAAGGAGTACCATATGATGAAAAATATTAA AGTCCGCACG	Construction of K1-Tar chimera
K1-Tar_R	CCACCAGCAGAATCAANNNNNNNNNNNNNNNCA TACAAACGCTGCACAGG	

Table S6. Microcalorimetric analysis of ligand binding to sensory domains from gut microbiota. For each protein, its affinities, experimental conditions, and enthalpy changes induced by the addition of relevant ligands were shown.

**Protein**			**Ligand**		**K_D (µM)**	**K_D Error (µM)**	**ΔH (kcal/mol)**	**ΔH Error (kcal/mol)**
**Pfam domain family**	**Protein ID**	**Concentration (µM)**	**Name**	**Concentration (mM)**				
SMP_2	B6	69	Indole	2	35.6	7.02	-6.2	1.5
sCache_2	K1	85	L-lactate	1	16.0	0.56	-26.9	0.5
		87	D-lactate	3	66.1	10.7	-5.41	0.5
		85	D-fructose	0.3	N/A	N/A	N/A	N/A
	C1	69	L-lactate	1	26.8	0.91	-51.0	1.7
dCache_1	B9	25	L-threonine	0.2	1.3	0.20	-19.5	1.1
		81	L-valine	1.5	N/A	N/A	N/A	N/A
	J6	73	Methylamine	2	242.0	22.00	-29.7	9.2
		73	Ethylamine	2	134.0	8.30	-55.1	16.3
	D8	63	Succinate	0.5	1.7	0.11	-21.9	0.3
		63	Maleate	0.5	7.7	0.41	-13.0	0.3
		63	Methylmalnote	1	28.0	1.90	-18.1	2.4
		63	Itaconate	0.3	9.6	0.90	-15.4	0.8
	H8	91	Uracil	0.3	4.6	0.33	-12.4	0.5
		91	Uridine	0.3	3.9	0.53	-10.8	0.7
		91	5-Fluorouracil	0.3	6.9	1.54	-17.3	2.3
		81	Acetate	2	103.0	4.72	-14.3	1.7
		81	Butyrate	2	133.0	13.10	-13.5	4.0
		81	Propionate	2	47.1	4.30	-10.5	1.0
	A4	87	Uracil	0.12	1.9	0.28	-25.8	2.9
		87	Acetate	0.5	50.3	5.76	-8.7	0.7
		87	Butyrate	0.5	29.9	2.36	-17.2	1.2
		72	Propionate	0.5	13.4	0.59	-12.2	0.2
	M8	70	Uracil	0.1	2.6	0.70	-39.3	12.6
		72	Butyrate	2	316.0	56.90	-31.3	80.6
		70	Propionate	8	633.0	104.00	-0.9	0.2
		70	Acetate	5	N/A	N/A	N/A	N/A

	M8-dm	78	Uracil	0.12	2.6	0.32	-23.6	0.9
		78	Butyrate	2	N/A	N/A	N/A	N/A
		78	Propionate	8	N/A	N/A	N/A	N/A
	Apo-A4	68	Uracil	0.7	$98.4x10^{-3}$	$6.6x10^{-3}$	-12.8	0.05
		67	Acetate	3	74.3	5.92	-9.9	1.14
		67	Butyrate	3	104.0	8.81	-5.5	0.57
		68	Propionate	3	16.2	0.47	-10.6	0.17
		67	Epinephrine	3	N/A	N/A	N/A	N/A
	Apo-A4_W160A	68	Uracil	3	106.0	13.00	-10.1	1.23
		68	Acetate	3	87.7	6.41	-6.8	0.51
	Apo-A4_D205A	66	Uracil	0.7	N/A	N/A	N/A	N/A
		66	Acetate	3	51.4	5.36	-4.6	0.31
	Apo-A4_Y225A	68	Uracil	0.7	$54.8x10^{-3}$	$8.9x10^{-3}$	-13.5	0.09
		68	Acetate	3	N/A	N/A	N/A	N/A
	Apo-A4_Y273A	68	Uracil	0.7	$99.3x10^{-3}$	$23.4x10^{-3}$	-6.2	0.09
		68	Acetate	3	N/A	N/A	N/A	N/A
	Apo-A4_R116A, T145A, N178A, N180G	67	Uracil	0.7	N/A	N/A	N/A	N/A
		67	Acetate	3	87.1	4.15	-11.5	0.83
		67	Epinephrine	3	169.0	24.50	-6.1	1.05
**Competitive ITC**	Apo-A4 with uracil	69	Acetate	3	63.7	4.85	-12.0	1.18
	Apo-A4 with acetate	66	Uracil	0.7	$75.1x10^{-3}$	$4.0x10^{-3}$	-14.0	0.04

Table S7. Crystallographic data collection and refinement statistics of A4 protein (PDB ID: 9HVJ).

**Data collection**	
Wavelength (Å)	0.87313
Resolution range	46.54 - 1.464 (1.516 - 1.464)
Space group	P 1 2_1 1
Unit cell	
a, b, c (Å)	35.00, 93.08, 44.38
α, β, γ (°)	90.0, 92.2, 90.0
Number of reflections	
Total	330,830 (30,918)
Unique	48,349 (4,659)
Multiplicity	6.8 (6.6)
Completeness (%)	99.07 (95.55)
Mean I/sigma(I)	13.29 (3.31)
Wilson B-factor	10.32
R-merge	0.1673 (0.9419)
R-meas	0.1812 (1.023)
R-pim	0.06886 (0.3939)
CC1/2	0.996 (0.732)
CC*	0.999 (0.919)
**Refinement**	
Number of used reflections	
Total	48,332 (4,659)
Free set	2,416 (233)
R-work	0.1681 (0.2338)
R-free	0.1890 (0.2699)
Number of atoms	2,579
Macromolecules	2,298
Ligands	16
Solvent	265
Average B-factor	15.83
Macromolecules	14.71
Ligands	11.18
Solvent	25.82
R.M.S. deviations	
Bonds lengths (Å)	0.007
Bond angles (°)	0.90
Ramachandran	
Favored (%)	97.85
Allowed (%)	2.15
Outliers (%)	0.00

Statistics for the highest-resolution shell are shown in parentheses.

Other supporting materials for this manuscript include the following:

Dataset S1 (separate file)**.** All studied extracytoplasmic sensory domains (LBD library).

Dataset S2 (separate file)**.** Bioinformatic analysis of sensors that contain the dCache_1UR motif and/or putative SCFA motif.

Dataset S3 (separate file)**.** Chemoreceptors that contain extracytoplasmic sensory domains in twelve gut bacteria.

SI References

1. W. Li *et al.*, The EMBL-EBI bioinformatics web and programmatic tools framework. *Nucleic Acids Res.* **43**, W580-584 (2015).
2. V. Zulkower, S. Rosser, DNA Chisel, a versatile sequence optimizer. *Bioinformatics* **36**, 4508-4509 (2020).
3. D. G. Gibson *et al.*, Enzymatic assembly of DNA molecules up to several hundred kilobases. *Nat. Methods* **6**, 343-345 (2009).
4. S. Bi, A. M. Pollard, Y. Yang, F. Jin, V. Sourjik, Engineering hybrid chemotaxis receptors in bacteria. *ACS Synth. Biol.* **5**, 989-1001 (2016).
5. W. Xu *et al.*, Systematic mapping of chemoreceptor specificities for *Pseudomonas aeruginosa*. *mBio* **14**, e0209923 (2023).
6. A. Paulick, V. Sourjik, FRET analysis of the chemotaxis pathway response. *Methods Mol. Biol.* **1729**, 107-126 (2018).
7. V. Sourjik, A. Vaknin, T. S. Shimizu, H. C. Berg, In vivo measurement by FRET of pathway activity in bacterial chemotaxis. *Methods Enzymol.* **423**, 365-365 (2007).
8. A. Velazquez-Campoy, G. Goñi, J. R. Peregrina, M. Medina, Exact analysis of heterotropic interactions in proteins: Characterization of cooperative ligand binding by isothermal titration calorimetry. *Biophys. J.* **91** (2006).
9. J. Abramson *et al.*, Accurate structure prediction of biomolecular interactions with AlphaFold 3. *Nature* **630**, 493-500 (2024).
10. H. M. Berman *et al.*, The protein data bank. *Acta Crystallogr. D Biol. Crystallogr.* **58** (2002).
11. E. C. Meng *et al.*, UCSF ChimeraX: Tools for structure building and analysis. *Protein Sci.* **32**, e4792 (2023).
12. S. Kim *et al.*, PubChem 2023 update. *Nucleic Acids Res.* **51**, D1373-D1380 (2023).
13. G. Corso, H. Stärk, B. Jing, R. Barzilay, T. Jaakkola, DiffDock: Diffusion steps, twists, and turns for molecular docking. *Preprint at https://doi.org/10.48550/arXiv.2210.01776.* (2022).
14. K. Katoh, D. M. Standley, MAFFT multiple sequence alignment software version 7: Improvements in performance and usability. *Mol. Biol. Evol.* **30** (2013).
15. A. M. Waterhouse, J. B. Procter, D. M. A. Martin, M. Clamp, G. J. Barton, Jalview Version 2-A multiple sequence alignment editor and analysis workbench. *Bioinformatics* **25** (2009).
16. S. Capella-Gutierrez, J. M. Silla-Martinez, T. Gabaldon, trimAl: a tool for automated alignment trimming in large-scale phylogenetic analyses. *Bioinformatics* **25**, 1972-1973 (2009).
17. D. Darriba, G. L. Taboada, R. Doallo, D. Posada, ProtTest 3: fast selection of best-fit models of protein evolution. *Bioinformatics* **27**, 1164-1165 (2011).
18. F. Ronquist *et al.*, MrBayes 3.2: efficient Bayesian phylogenetic inference and model choice across a large model space. *Syst. Biol.* **61**, 539-542 (2012).
19. J. Koblitz *et al.*, MediaDive: the expert-curated cultivation media database. *Nucleic Acids Res.* **51**, D1531-D1538 (2023).
20. N. T. Wirth, J. Funk, S. Donati, P. I. Nikel, QurvE: user-friendly software for the analysis of biological growth and fluorescence data. *Nat. Protoc.* **18**, 2401-2403 (2023).
21. W. Kabsch, Integration, scaling, space-group assignment and post-refinement. *Acta Crystallogr. D Biol. Crystallogr.* **66**, 133-144 (2010).
22. A. J. McCoy *et al.*, Phaser crystallographic software. *J. Appl. Crystallogr.* **40**, 658-674 (2007).

23. P. Emsley, B. Lohkamp, W. G. Scott, K. Cowtan, Features and development of Coot. *Acta Crystallogr. D Biol. Crystallogr.* **66**, 486-501 (2010).
24. D. Liebschner *et al.*, Macromolecular structure determination using X-rays, neutrons and electrons: recent developments in Phenix. *Acta Crystallogr. D Struct. Biol.* **75**, 861-877 (2019).
25. R. A. Laskowski, J. Jablonska, L. Pravda, R. S. Varekova, J. M. Thornton, PDBsum: Structural summaries of PDB entries. *Protein Sci.* **27**, 129-134 (2018).
26. S. Broer, Intestinal amino acid transport and metabolic health. *Annu. Rev. Nutr.* **43**, 73-99 (2023).
27. E. A. Smith, G. T. Macfarlane, Studies on amine production in the human colon: Enumeration of amine forming bacteria and physiological effects of carbohydrate and pH. *Anaerobe* **2**, 285-297 (1996).
28. L. F. Mager *et al.*, Microbiome-derived inosine modulates response to checkpoint inhibitor immunotherapy. *Science* **369**, 1481-1489 (2020).
29. K. H. U. Meier *et al.*, Metabolic landscape of the male mouse gut identifies different niches determined by microbial activities. *Nat. Metab.* **5**, 968-980 (2023).
30. T. A. Psarra, G. C. Batzias, T. L. Peeters, M. Koutsoviti-Papadopoulou, The gastrointestinal effects that may follow the administration of theophylline reflect the pharmacodynamic profiles of both the parent drug and its metabolites. *Fundam. Clin. Pharmacol.* **24**, 171-180 (2010).
31. J. Connors, N. Dawe, J. Van Limbergen, The role of succinate in the regulation of intestinal inflammation. *Nutrients* **11** (2018).
32. E. R. Hughes *et al.*, Microbial respiration and formate oxidation as metabolic signatures of inflammation-associated dysbiosis. *Cell Host Microbe* **21**, 208-219 (2017).
33. B. Kircher *et al.*, Predicting butyrate- and propionate-forming bacteria of gut microbiota from sequencing data. *Gut Microbes* **14**, 2149019 (2022).
34. G. Gargari, V. Taverniti, R. Koirala, C. Gardana, S. Guglielmetti, Impact of a multistrain probiotic formulation with high bifidobacterial content on the fecal bacterial community and short-chain fatty acid levels of healthy adults. *Microorganisms* **8** (2020).
35. J. Xie *et al.*, Fructose metabolism and its role in pig production: A mini-review. *Front. Nutr.* **9**, 922051 (2022).
36. J. Yang *et al.*, Biphasic chemotaxis of *Escherichia coli* to the microbiota metabolite indole. *Proc. Natl Acad. Sci. USA* **117**, 6114-6120 (2020).
37. B. P. Anton, A. Fomenkov, E. A. Raleigh, M. Berkmen, Complete genome sequence of the engineered *Escherichia coli* SHuffle strains and their wild-type parents. *Genome Announc.* **4** (2016).
38. D. M. Woodcock *et al.*, Quantitative evaluation of *Escherichia coli* host strains for tolerance to cytosine methylation in plasmid and phage recombinants. *Nucleic Acids Res.* **17**, 3469-3478 (1989).
39. P. Ames, C. A. Studdert, R. H. Reiser, J. S. Parkinson, Collaborative signaling by mixed chemoreceptor teams in *Escherichia coli*. *Proc. Natl Acad. Sci. USA* **99**, 7060-7065 (2002).
40. V. Sourjik, H. C. Berg, Functional interactions between receptors in bacterial chemotaxis. *Nature* **428**, 437-441 (2004).
41. M. D. C. Burón-Barral, K. K. Gosink, J. S. Parkinson, Loss- and gain-of-function mutations in the F1-HAMP region of the *Escherichia coli* aerotaxis transducer Aer. *J. Bacteriol.* **188**, 3477-3486 (2006).
42. S. Bi, F. Jin, V. Sourjik, Inverted signaling by bacterial chemotaxis receptors. *Nat. Commun.* **9**, 2927 (2018).
